# Supplementary material for: The impact of community based interventions for the prevention and control of soil-transmitted helminths: A systematic review and meta-analysis
Source: PLOS Glob Public Health. 2024 Oct 10;4(10):e0003717. doi: 10.1371/journal.pgph.0003717 (PMC11466416; doi:10.1371/journal.pgph.0003717)
Supplement: S1 Data — Table A. PRIMARY OUTCOME DATA EXTRACTED FROM INCLUDED STUDIES Data from each included study arranged into subtables. Row 1 of each subtable outlines the study citation. Row 2 of each subtable specifies the follow-up period. Row 3 of each subtable specifies the study design. Row 4 of each subtable outlines the intervention type. Subsequent rows report the prevalence at baseline and endline for Hookworm, A. lumbricoided and T. trichiura where available. Infection intensity and nutritional outcomes are also reported where available. Table B. QUALITY ASSESSMENT SUMMARY TABLE Domains D1 to D7 corresponds to the domains outlined in the Cochrane risk of bias tool (RoB2) and the risk of bias in non-randomized studies—of Interventions tool (ROBINS-I) (see references [12 & 13]). Table C. FULL SEARCH TERMS USED Column 1: Key search terms Column 2: Synonyms used in database search. (DOCX) [file pgph.0003717.s002.docx]

**Table A. PRIMARY OUTCOME DATA EXTRACTED FROM INCLUDED STUDIES**

| Ash, A., Okello, A., Khamlome, B., Inthavong, P., Allen, J., & Thompson, R. A. (2017). Controlling Taenia solium and soil transmitted helminths in a northern Lao PDR village: Impact of a triple dose albendazole regime. Acta tropica, 174, 171-178. | | | | | |
| --- | --- | --- | --- | --- | --- |
| Data Extractor | | | Michael Muoka | | |
| Date of Data extraction | | | 23^rd^ January, 2024 | | |
| Confirmation of Eligibility | | | Data on the effectiveness of CBI for STHs reported; baseline and endline data reported; specie specific data reported | | |
| Follow-up | | | 16 months | | |
| Design | | | before-after study | | |
| Intervention | | | triple dose albendazole 400 mg (Eskazole®, GlaxoSmithKline) delivered over three consecutive days | | |
|  | Prevalence at Baseline | Prevalence at Endline | | Avg EPG at Baseline | Avg EPG at Endline |
| **Hookworm** | 56% (n = 56) | 4.2% (n = 2) | | 233 | 5.3 |
| **A. lumbricoides** | 43% (n = 43) | 2.1% (n = 1) | | 2818 | 16.6 |
| **T. trichiura** | 60% (n = 60) | 16.7%(n = 8) | | 166.5 | 14.6 |
|  |  |  | |  |  |

| Al-Delaimy, A. K., Al-Mekhlafi, H. M., Lim, Y. A., Nasr, N. A., Sady, H., Atroosh, W. M., & Mahmud, R. (2014). Developing and evaluating health education learning package (HELP) to control soil-transmitted helminth infections among Orang Asli children in Malaysia. Parasites & vectors, 7, 416. https://doi.org/10.1186/1756-3305-7-416 | | | | | | | |
| --- | --- | --- | --- | --- | --- | --- | --- |
| Data Extractors | | | | Michael Muoka | | | |
| Date of Data extraction | | | | 23^rd^ January, 2024 | | | |
| Confirmation of Eligibility | | | | Data on the effectiveness of CBI for STHs reported; baseline and endline data reported; specie specific data reported | | | |
| Follow-up timeline | | | | 6 months | | | |
| Design | | | | open-label controlled intervention trial | | | |
| Intervention | | | | health education learning package (HELP) | | | |
| Sample Group | **Hookworm** | **T. trichiura** | **A. lumbricoides** | | Infection intensity (**T. trichiura)** | Infection intensity (**Hookworm**) | Infection intensity (**A. lumbricoides**) |
| Baseline HELP (172) | 63 (36.6%) | 164 (95.3%) | 90 (52.3%) | | 6327.45 | 276.67 | 11075.48 |
| Baseline Control (145) | 45 (31.0%) | 141 (97.2%) | 73 (50.3%) | | 6218.79 | 241.82 | 10893.37 |
| Endline HELP (172) | not given (14.8%) | not given (75.5%) | not given (39.6%) | | 3875.01 | 79.98 | 6266.57 |
| Endline Control (145) | not given (23.7%) | not given (82.3%) | not given (41.6%) | | 5013.82 | 227.35 | 9821.12 |

| Al-Mekhlafi, H. M., Anuar, T. S., Al-Zabedi, E. M., Al-Maktari, M. T., Mahdy, M. A., Ahmed, A., Sallam, A. A., Abdullah, W. A., Moktar, N., & Surin, J. (2014). Does vitamin A supplementation protect schoolchildren from acquiring soil-transmitted helminthiasis? A randomized controlled trial. Parasites & vectors, 7, 367. https://doi.org/10.1186/1756-3305-7-367 | | | | | | | |
| --- | --- | --- | --- | --- | --- | --- | --- |
| Data Extractors | | | | Michael Muoka | | | |
| Date of Data extraction | | | | 23^rd^ January, 2024 | | | |
| Confirmation of Eligibility | | | | Data on the effectiveness of CBI for STHs reported; baseline and endline data reported; specie specific data reported | | | |
| Follow-up timeline | | | | 6 months | | | |
| Design | | | | randomized, double-blind, placebo-controlled trial | | | |
| Intervention | | | | High dose vitamin A | | | |
| Sample Group (N) | **Hookworm** | **T. trichiura** | **A. lumbricoides** | | Infection intensity (**T. trichiura)** | Infection intensity (**Hookworm**) | Infection intensity (**A. lumbricoides**) |
| Baseline Vit A (121) | (14.9%) | (96.7%) | (69.4%) | | 5,474 (2,031) | 59 (20) | 19,869 (9,744) |
| Baseline Control (120) | (10.8%) | (97.5%) | (65.8%) | | 5,624 (3,449) | 57 (19) | 16,072 (8,151) |
| Endline Vit A (108) | (56.4%) | (65.8%) | (76.8%) | | 2,859 (799) | 14 (11) | 14,867 (5,246) |
| Endline Control (106) | (51.9%) | (66.5%) | (73.1%) | | 3,678 (562) | 11 (8) | 13,368 (6,367) |

| Chen, Y. D., Li, H. Z., Xu, L. Q., Qian, M. B., Tian, H. C., Fang, Y. Y., Zhou, C. H., Ji, Z., Feng, Z. J., Tang, M., Li, Q., Wang, Y., Bergquist, R., & Zhou, X. N. (2021). Effectiveness of a community-based integrated strategy to control soil-transmitted helminthiasis and clonorchiasis in the People's Republic of China. Acta tropica, 214, 105650. https://doi.org/10.1016/j.actatropica.2020.105650 | |
| --- | --- |
| Data Extractors | Michael Muoka |
| Date of Data extraction | 23^rd^ January, 2024 |
| Confirmation of Eligibility | Data on the effectiveness of CBI for STHs reported; specie specific data reported |
| Follow-up timeline | yearly |
| Design | cross-sectional survey |
| Intervention; SBT | Integrated MDA + Health education + Sanitation |
| Reported results | The reduction rate of A. lumbricoides infection from 2006 to 2009 ranged from 63.8% to 99.0%, with an average of 85.8% |
|  | The reduction of hookworm infections from 2006 to 2009 ranged from 59.0% to 96.7%, with an average of 76.6%. |
|  | The reduction rate of T. trichiura infection from 2006 to 2009 ranged from 65.8% to 90.0%, with an average of 79.6%. |

| Muslim, A., & Lim, Y. A. L. (2022). RESEARCH ARTICLE Higher efficacy of a single dosage albendazole and different soil-transmitted helminths re-infection profiles amongst indigenous Negritos from inland jungle versus those in resettlement at town peripheries. Tropical Biomedicine, 39(3), 402-411. | | | | | | | |
| --- | --- | --- | --- | --- | --- | --- | --- |
| Data Extractors | | | | Michael Muoka | | | |
| Date of Data extraction | | | | 23^rd^ January, 2024 | | | |
| Confirmation of Eligibility | | | | Data on the effectiveness of CBI for STHs reported; baseline and endline data reported; specie specific data reported | | | |
| Follow-up timeline | | | | 6 months | | | |
| Design | | | | longitudinal follow-up study | | | |
| Intervention | | | | CWT; supervised mass albendazole treatment | | | |
| Sample Group | **Hookworm** | **T. trichiura** | **A. lumbricoides** | | Infection intensity (**T. trichiura)** | Infection intensity (**Hookworm**) | Infection intensity (**A. lumbricoides**) |
| pre-treatment | 10 (18.5%) | 38 (70.4%) | 27 (50.0%) | | not given | not given | not given |
| Endline 6mo | 5 (11.6%) | 41 (95.3%) | 21 (48.8%) | | not given | not given | not given |

| Vaz Nery, S., Traub, R. J., McCarthy, J. S., Clarke, N. E., Amaral, S., Llewellyn, S., Weking, E., Richardson, A., Campbell, S. J., Gray, D. J., Vallely, A. J., Williams, G. M., Andrews, R. M., & Clements, A. C. A. (2019). WASH for WORMS: A Cluster-Randomized Controlled Trial of the Impact of a Community Integrated Water, Sanitation, and Hygiene and Deworming Intervention on Soil-Transmitted Helminth Infections. The American journal of tropical medicine and hygiene, 100(3), 750–761. https://doi.org/10.4269/ajtmh.18-0705 | | | | | | | |
| --- | --- | --- | --- | --- | --- | --- | --- |
| Data Extractors | | | | Michael Muoka | | | |
| Date of Data extraction | | | | 25^th^ January, 2024 | | | |
| Confirmation of Eligibility | | | | Data on the effectiveness of CBI for STHs reported; baseline and endline data reported; specie specific data reported | | | |
| Follow-up timeline | | | | 24 months (6 monthly follow-up) | | | |
| Design | | | | Cluster RCT | | | |
| Intervention | | | | WASH | | | |
| Sample Group (N) | **Hookworm** | **T. trichiura** | **A. lumbricoides** | | Infection intensity (**T. trichiura)** | Infection intensity (**Hookworm**) | Infection intensity (**A. lumbricoides**) |
| baseline WASH (901) | 453 (63.7%) | 5 (0.7%) | 156 (21.9%) | | Not given | Not given | Not given |
| Baseline control (1,046) | 576 (64.6%) | 1 (0.1%) | 125 (14.0%) | | Not given | Not given | Not given |
| Endline WASH (553) | 15.4 (9.6−24.6) | Not given | 14.3% (2.9−30.3) | | Not given | Not given | Not given |
| Endline control (623) | 16.9 (11.6−28.2) | Not given | 4.5% (0.0−13.3) | | Not given | Not given | Not given |

| Sunish, I. P., Rajendran, R., Munirathinam, A., Kalimuthu, M., Kumar, V. A., Nagaraj, J., & Tyagi, B. K. (2015). Impact on prevalence of intestinal helminth infection in school children administered with seven annual rounds of diethyl carbamazine (DEC) with albendazole. The Indian journal of medical research, 141(3), 330–339. https://doi.org/10.4103/0971-5916.156622 | | | | | | | |
| --- | --- | --- | --- | --- | --- | --- | --- |
| Data Extractors | | | | Michael Muoka | | | |
| Date of Data extraction | | | | 25^th^ January, 2024 | | | |
| Confirmation of Eligibility | | | | Data on the effectiveness of CBI for STHs reported; baseline and endline data reported; specie specific data reported | | | |
| Follow-up timeline | | | | 24 months (6 monthly follow-up) | | | |
| Design | | | | before-after study | | | |
| Intervention | | | | MDA (DEC+ALB/ DEC only) | | | |
| Sample Group | **Hookworm** | **T. trichiura** | **A. lumbricoides** | | Infection intensity (**T. trichiura)** | Infection intensity (**Hookworm**) | Infection intensity (**A. lumbricoides**) |
| baseline (DEC + ALB) | 16.51% | 4.67% | 54.83% | | Not given | Not given | Not given |
| Endline (DEC + ALB) | 1.33% | 0.57% | 4.19% | | Not given | Not given | Not given |

| Landeryou, T., Maddren, R., Rayment Gomez, S., Kalahasti, S., Liyew, E. F., Chernet, M., ... & Anderson, R. (2022). Longitudinal monitoring of prevalence and intensity of soil-transmitted helminth infections as part of community-wide mass drug administration within the Geshiyaro project in the Bolosso Sore district, Wolaita, Ethiopia. PLoS Neglected Tropical Diseases, 16(9), e0010408. | | | | | | | |
| --- | --- | --- | --- | --- | --- | --- | --- |
| Data Extractors | | | | Michael Muoka | | | |
| Date of Data extraction | | | | 25^th^ January, 2024 | | | |
| Confirmation of Eligibility | | | | Data on the effectiveness of CBI for STHs reported; baseline and endline data reported; specie specific data reported | | | |
| Follow-up timeline | | | | 2 years | | | |
| Design | | | | longitudinal study | | | |
| Intervention | | | | Mass drug administration | | | |
| Sample Group (N) | **Hookworm** | **T. trichiura** | **A. lumbricoides** | | Infection intensity (**T. trichiura)** | Infection intensity (**Hookworm**) | Infection intensity (**A. lumbricoides**) |
| Baseline (143) | 4.38% | 6.08% | 29.7% | | 70.2 | 9.21 | 657 |
| Y1 Survey (170) | 16.4% | 9.59% | 22.1% | | 69.92 | 18.87 | 871 |
| Y2 Survey (127) | 9.98% | 2.9% | 25.9% | | 1.1 | 5.03 | 328 |

| Le, B., Clarke, N. E., Hii, S. F., Byrne, A., Khattak, A., Lake, S., ... & Nery, S. V. (2023). Effectiveness of one and two doses of ivermectin mass drug administration in reducing the prevalence and intensity of soil-transmitted helminth (STH) infections in Western Province, Solomon Islands: a cluster-randomised, before-after analysis. The Lancet Regional Health–Western Pacific. | | | | | | | |
| --- | --- | --- | --- | --- | --- | --- | --- |
| Data Extractors | | | | Michael Muoka | | | |
| Date of Data extraction | | | | 25^th^ January, 2024 | | | |
| Confirmation of Eligibility | | | | Data on the effectiveness of CBI for STHs reported; baseline and endline data reported; specie specific data reported | | | |
| Follow-up timeline | | | | 21 months | | | |
| Design | | | | RCT | | | |
| Intervention | | | | Mass drug administration | | | |
| Sample Group (N) | **Hookworm %** | **T. trichiura %** | **A. lumbricoides %** | | Infection intensity (**T. trichiura)** | Infection intensity (**Hookworm**) | Infection intensity (**A. lumbricoides**) |
| Baseline (IVM single, 372) | 51.6 (180) 46.5–56.7  19.9 (74) 16.1–24.3 | 19.9 (74) 16.1–24.3 | 0.27 (1) 0.04–1.9 | | 253 (794) | 754 (1526) | 33,713 (0) |
| Endline (IVM single, 505) | 52.3 (241) 47.9–56.6  18.8 (95) 15.6–22.5 | 11.1 (56) 8.6–14.1 | 0.6 (3) 0.2–1.8 | | 65.8 (101) | 924 (1938) | 46.5 (37.6) |
| baseline (IVM double, 458) | 58.5 (268) 53.4–63.0  14.4 (66) 11.5–17.9 | 21.4 (98) 17.9–25.4 | 1.1 (5) 0.5–2.6 | | 648 (1484) | 1040 (2010) | 58,375 (130,527) |
| Endline (IVM double, 674) | 61.3 (413) 57.5–64.9  19.6 (132) 16.8–22.8 | 12.0 (81) 9.8–14.7 | 1.3 (9) 0.7–2.5 | | 102 (147) | 854 (1925) | 7232 (11,847) |

| Le, B., Monteiro, M. A. A., Amaral, S., Wand, H., Matthews, A., Hii, S. F., ... & Nery, S. V. (2023). The impact of ivermectin, diethylcarbamazine citrate, and albendazole mass drug administration on the prevalence of scabies and soil-transmitted helminths in school-aged children in three municipalities in Timor-Leste: a before–after assessment. The Lancet Global Health, 11(6), e924-e932. | | | | | | | |
| --- | --- | --- | --- | --- | --- | --- | --- |
| Data Extractors | | | | Michael Muoka | | | |
| Date of Data extraction | | | | 25^th^ January, 2024 | | | |
| Confirmation of Eligibility | | | | Data on the effectiveness of CBI for STHs reported; baseline and endline data reported; specie specific data reported | | | |
| Follow-up timeline | | | | 18 months | | | |
| Design | | | | Before-after study | | | |
| Intervention | | | | Mass drug administration (IVM + DEC +ALB) | | | |
| Sample Group | **Hookworm** | **T. trichiura** | **A. lumbricoides** | | Infection intensity (**T. trichiura)** | Infection intensity (**Hookworm**) | Infection intensity (**A. lumbricoides**) |
| Baseline | 44/541 | 26/541 | 98/541 | | 130 (296) | 456 (817) | 20,083 (35,288) |
| Endline | 58/623 | 4/623 | 82/623 | | 77.7 (94.0) | 514 (1011) | 17,975 (40,195) |

| Gyorkos, T. W., Maheu-Giroux, M., Blouin, B., & Casapia, M. (2013). Impact of health education on soil-transmitted helminth infections in schoolchildren of the Peruvian Amazon: a cluster-randomized controlled trial. PLoS neglected tropical diseases, 7(9), e2397. | | | | | | | |
| --- | --- | --- | --- | --- | --- | --- | --- |
| Data Extractors | | | | Michael Muoka | | | |
| Date of Data extraction | | | | 25^th^ January, 2024 | | | |
| Confirmation of Eligibility | | | | Data on the effectiveness of CBI for STHs reported; baseline and endline data reported; specie specific data reported | | | |
| Follow-up timeline | | | | 4 months | | | |
| Design | | | | Cluster-randomised controlled trial | | | |
| Intervention | | | | School based health-education package | | | |
| Sample Group (N) | **Hookworm** | **T. trichiura** | **A. lumbricoides** | | Infection intensity (**T. trichiura)** | Infection intensity (**Hookworm**) | Infection intensity (**A. lumbricoides**) |
| Baseline Intervention (518) | 12.0% | 59.1% | 49.2% | | 1045 (2841) | 43 (194) | 8456 (17744) |
| Endline Intervention (518) | 6.6% | 52.1% | 31.9% | | 450.6 (1659) | 11.2 (70) | 1392 (5927) |
| Baseline Control (571) | 12.6% | 64.8% | 55.5% | | 832 (2344) | 52 (417) | 9968 (22020) |
| Endline Control (571) | 4.9% | 56.7% | 36.4% | | 309.8 (760) | 7.9 (73) | 2147 (7206) |

| Dunn JC, Bettis AA, Wyine NY, Lwin AMM, Tun A, Maung NS, Anderson RM. Soil-transmitted helminth reinfection four and six months after mass drug administration: results from the delta region of Myanmar. PLoS Negl Trop Dis. 2019 Feb 15;13(2):e0006591. doi: 10.1371/journal.pntd.0006591. | |
| --- | --- |
| Data Extractors | Michael Muoka |
| Date of Data extraction | 25^th^ January, 2024 |
| Confirmation of Eligibility | Data on the effectiveness of CBI for STHs reported; baseline and endline data reported; specie specific data reported |
| Follow-up timeline | 11 months |
| Design | Longitudinal study |
| Intervention | Albendazole/ diethylcarbamazine citrate |

|  | **Any STH** | | ***Ascaris lumbricoides*** | | | ***Trichuris trichiura*** | | | **Hookworm** | | |
| --- | --- | --- | --- | --- | --- | --- | --- | --- | --- | --- | --- |
|  | **n** | **% (95% CI)^*^** | **n** | **% (95% CI)** | **Mean EPG (95% CI)** | **n** | **% (95% CI)** | **Mean EPG (95% CI)** | **n** | **% (95% CI)** | **Mean EPG (95% CI)** |
| **Survey 1 (Aug 2015)** | 146 | 27.92 (24.64–32.64) | 29 | 5.54 (3.83–8.03) | 649.42 (370.35–1119.4) | 89 | 17.02 (14.2–20.95) | 73.56 (46.99–124.35) | 51 | 9.75 (7.51–12.89) | 40.2 (22.39–93.3) |
| **Survey 2 (Dec 2015)** | 99 | 18.93 (16.00–23.03) | 9 | 1.72 (0.81–3.31) | 478.12 (87.36–1967.6) | 62 | 11.85 (9.41–15.25) | 24.32 (13.77–54.47) | 35 | 6.69 (4.81–9.38) | 314.94 (10.69–1533.86) |
| **Survey 3 (Jun 2016)** | 99 | 18.93 (16.00–23.03) | 13 | 2.49 (1.36–4.3) | 670.35 (315.2–1392.34) | 62 | 11.85 (9.41–15.25) | 41.07 (24.36–72.28) | 29 | 5.54 (3.83–8.03) | 11.47 (6.38–24.78) |

| Freeman, M. C., Clasen, T., Brooker, S. J., Akoko, D. O., & Rheingans, R. (2013). The impact of a school-based hygiene, water quality and sanitation intervention on soil-transmitted helminth reinfection: a cluster-randomized trial. The American journal of tropical medicine and hygiene, 89(5), 875–883. https://doi.org/10.4269/ajtmh.13-0237 | | | | | | | |  |
| --- | --- | --- | --- | --- | --- | --- | --- | --- |
| Data Extractors | | | | Sommy Ugwu | | | |  |
| Date of Data extraction | | | | 17^th^ August, 2022 | | | |  |
| Confirmation of Eligibility | | | | Data on the effectiveness of CBI for STHs reported; baseline and endline data reported; specie specific data reported | | | |  |
| Follow-up timeline | | | | 20 months | | | |  |
| Design | | | | cRCT | | | |  |
| Intervention | | | | WASH | | | |  |
| Sample Group (N) | **Hookworm** | **T. trichiura** | **A. lumbricoides** | | Infection intensity (**T. trichiura)** | Infection intensity (**Hookworm**) | Infection intensity (**A. lumbricoides**) | |
| Baseline Intervention (470) | 29.4 (19.0) | 5.9 (7.2) | 14.2 (13.9) | | 13.7 (31.1) | 145.4 (153.1) | 1094 (1488) | |
| Endline Intervention | 8.8 (7.2) | 7.3 (6.1) | 6.2 (6.9) | | 23.0 (70.5) | 34.4 (48.7) | 395 (623) | |
| Baseline Control (445) | 27.7 (21.2) | 4.4 (4.9) | 9.2 (14.2) | | 7.1 (11.6) | 104.1 (164.1) | 506 (1049) | |
| Endline Control | 5.4 (5.8) | 6.5 (6.5) | 8.6 (14.6) | | 33.1 (62.0) | 31.8 (54.1) | 796 (1337) | |

| Pion, S. D., Chesnais, C. B., Bopda, J., Louya, F., Fischer, P. U., Majewski, A. C., Weil, G. J., Boussinesq, M., & Missamou, F. (2015). The impact of two semiannual treatments with albendazole alone on lymphatic filariasis and soil-transmitted helminth infections: a community-based study in the Republic of Congo. The American journal of tropical medicine and hygiene, 92(5), 959–966. https://doi.org/10.4269/ajtmh.14-0661 | | | | | | | |
| --- | --- | --- | --- | --- | --- | --- | --- |
| Data Extractors | | | | Sommy Ugwu | | | |
| Date of Data extraction | | | | 17^th^ August, 2022 | | | |
| Confirmation of Eligibility | | | | Data on the effectiveness of CBI for STHs reported; baseline and endline data reported; specie specific data reported | | | |
| Follow-up timeline | | | | 1 year | | | |
| Design | | | | Before-after study | | | |
| Intervention | | | | Albendazole | | | |
| Sample Group (N) | **Hookworm** | **T. trichiura** | **A. lumbricoides** | | Infection intensity (**T. trichiura)** | Infection intensity (**Hookworm**) | Infection intensity (**A. lumbricoides**) |
| Baseline (462) | 6.5 [4.2–8.7] | 78.6 [74.8–82.3] | 56.4 [52.0–61.0] | | 1,409.5 [1125.7–1693.2] | 67.6 [33.7–101.5] | 17,426.0 [14,884.4–19,967.6] |
| Endline (335) | 0.6 [0.0–1.4] | 71.0 [66.2–75.9] | 36.4 [31.2–41.6] | | 1,034.2 [623.5–1444.9] | 18.0 [0.0–94.2] | 4,083.8 [2,862.1–5,827.8] |

| Okoyo, C., Nikolay, B., Kihara, J., Simiyu, E., Garn, J. V., Freeman, M. C., ... & Mwandawiro, C. S. (2016). Monitoring the impact of a national school based deworming programme on soil-transmitted helminths in Kenya: the first three years, 2012–2014. Parasites & vectors, 9, 1-13. | | | | | | | |
| --- | --- | --- | --- | --- | --- | --- | --- |
| Data Extractors | | | | Sommy Ugwu | | | |
| Date of Data extraction | | | | 17^th^ August, 2022 | | | |
| Confirmation of Eligibility | | | | Data on the effectiveness of CBI for STHs reported; baseline and endline data reported; specie specific data reported | | | |
| Follow-up timeline | | | | 3 years | | | |
| Design | | | | Before-after study | | | |
| Intervention | | | | Albendazole | | | |
| Sample Group (N) | **Hookworm** | **T. trichiura** | **A. lumbricoides** | | Infection intensity (**T. trichiura)** | Infection intensity (**Hookworm**) | Infection intensity (**A. lumbricoides**) |
| Baseline (21,432) | 15.4 (13.6–17.6) | 6.7 (5.4–8.2) | 18.1 (15.8–20.7) | | 33 (11–105) | 63 (50–81) | 1659 (1378–1998) |
| Endline (21,011) | 2.3 (1.8–3.0) | 4.5 (3.4–6.0) | 11.9 (10.2–13.9) | | 17 (11–26) | 8 (5–14) | 960 (801–1151) |

| Echazú A, Juarez M, Vargas PA, Cajal SP, Cimino RO, Heredia V, et al. (2017) Albendazole and ivermectin for the control of soil-transmitted helminths in an area with high prevalence of Strongyloides stercoralis and hookworm in northwestern Argentina: A community-based pragmatic study. PLoS Negl Trop Dis 11(10): e0006003. https://doi.org/10.1371/journal.pntd.0006003 | | | | | | | |
| --- | --- | --- | --- | --- | --- | --- | --- |
| Data Extractors | | | | Michael Muoka | | | |
| Date of Data extraction | | | | 26^th^ January, 2024 | | | |
| Confirmation of Eligibility | | | | Data on the effectiveness of CBI for STHs reported; baseline and endline data reported; specie specific data reported | | | |
| Follow-up timeline | | | | 3 years | | | |
| Design | | | | Community-based pragmatic non-randomized trial | | | |
| Intervention | | | | Albendazole + IVM | | | |
| Sample Group (N) | **Hookworm** | **T. trichiura** | **A. lumbricoides** | | Infection intensity (**T. trichiura)** | Infection intensity (**Hookworm**) | Infection intensity (**A. lumbricoides**) |
| Baseline | 93/197 | 3/397 | 15/397 | | Not given | Not given | Not given |
| Endline | 20/181 | Not given | Not given | | Not given | Not given | Not given |
|  |  |  |  | |  |  |  |
|  | Stunting | Underweight | Wasting | | Anaemia |  |  |
| Baseline | 118/613 | 16/456 | 4/198 | | 84/151 |  |  |
| Endline | 46/368 | 12/297 | 4/113 | | 24/165 |  |  |

| Pion, S. D., Chesnais, C. B., Weil, G. J., Fischer, P. U., Missamou, F., & Boussinesq, M. (2017). Effect of 3 years of biannual mass drug administration with albendazole on lymphatic filariasis and soil-transmitted helminth infections: a community-based study in Republic of the Congo. The Lancet infectious diseases, 17(7), 763-769. | | | | | | | |
| --- | --- | --- | --- | --- | --- | --- | --- |
| Data Extractors | | | | Sommy Ugwu | | | |
| Date of Data extraction | | | | 17^th^ August, 2022 | | | |
| Confirmation of Eligibility | | | | Data on the effectiveness of CBI for STHs reported; baseline and endline data reported; specie specific data reported | | | |
| Follow-up timeline | | | | 3 years | | | |
| Design | | | | Before-after | | | |
| Intervention | | | | Albendazole | | | |
| Sample Group (N) | **Hookworm** | **T. trichiura** | **A. lumbricoides** | | Infection intensity (**T. trichiura)** | Infection intensity (**Hookworm**) | Infection intensity (**A. lumbricoides**) |
| Baseline (462) | 6·5% (4·2–8·7) | 78·6% (74·8–82·3) | 56·5% (52·0–61·0) | | 1107·4 (878·5–1336·3) | 4·4 (1·8–7·0) | 9844·6 (8209·0–11 480·0) |
| Endline (350) | 0 | 59·4% (54·2–64·5) | 12·9% (9·7–16·8) | | 366·0 (255·7–476·2) | 0 | 724·4 (340·7–1114·2) |

| Paige, S. B., Friant, S., Clech, L., Malavé, C., Kemigabo, C., Obeti, R., & Goldberg, T. L. (2017). Combining Footwear with Public Health Iconography to Prevent Soil-Transmitted Helminth Infections. The American journal of tropical medicine and hygiene, 96(1), 205–213. https://doi.org/10.4269/ajtmh.15-0910 | | | | | | | |
| --- | --- | --- | --- | --- | --- | --- | --- |
| Data Extractors | | | | Sommy Ugwu | | | |
| Date of Data extraction | | | | 18^th^ August, 2022 | | | |
| Confirmation of Eligibility | | | | Data on the effectiveness of CBI for STHs reported; baseline and endline data reported; specie specific data reported | | | |
| Follow-up timeline | | | | 6 months | | | |
| Design | | | | Before-after | | | |
| Intervention | | | | Shoes + Iconography | | | |
| Sample Group (N) | **Hookworm** | **T. trichiura** | **A. lumbricoides** | | Infection intensity (**T. trichiura)** | Infection intensity (**Hookworm**) | Infection intensity (**A. lumbricoides**) |
| Baseline Control (75) | 0.19 (0.12–0.29) | 0.16 (0.09–0.26 | 0.37 (0.26–0.49) | | 37.2 (15.5–90.1) | 16 (8.64–26.1) | 797 (307–1,850) |
| Endline Control (71) | 0.06 (0.02–0.14) | 0.16 (0.08–0.26) | 0.28 (0.19–0.40 | | 16.9 (10.3–28.6) | 6.25 (2–12.5) | 760 (281–2,100) |
| Baseline EXP 1(99) | 0.07 (0.03–0.14) | 0.22 (0.15–0.32) | 0.07 (0.03–0.14) | | 17.3 (11.4–26.4) | 36.7 (6.71–121) | 126 (72.1–273) |
| Endline EXP 1(67) | 0.06 (0.02–0.15) | 0.12 (0.05–0.22) | 0.09 (0.03–0.19) | | 20.8 (4.38–49.1) | 1.5 (1–2) | 203 (57.2–566) |
| Baseline EXP 2(50) | 0.14 (0.06–0.27) | 0.02 (0.00–0.11) | 0.10 (0.03–0.22) | | 2 | 23.7 (7.29–62.3) | 34 (2.2–75) |
| Endline EXP 1(48) | 0.17 (0.07–0.30) | 0.02 (0.00–0.11) | 0.02 (0.00–0.11) | | 3 (NA) | 7 (3.75–10.8) | 85 (NA) |
| Experimental 1: given Holoflops; Experimental 2: given plain shoes; Control: no shoes distributed during the study period. | | | | | | | |
| Clarke, N. E., Clements, A. C. A., Amaral, S., Richardson, A., McCarthy, J. S., McGown, J., Bryan, S., Gray, D. J., & Nery, S. V. (2018). (S)WASH-D for Worms: A pilot study investigating the differential impact of school- versus community-based integrated control programs for soil-transmitted helminths. PLoS neglected tropical diseases, 12(5), e0006389. https://doi.org/10.1371/journal.pntd.0006389 | | | | | | | |
| Data Extractors | | | | Michael Muoka | | | |
| Date of Data extraction | | | | 26^th^ January, 2024 | | | |
| Confirmation of Eligibility | | | | Data on the effectiveness of CBI for STHs reported; baseline and endline data reported; specie specific data reported | | | |
| Follow-up timeline | | | | 6 months | | | |
| Design | | | | non-randomized cluster intervention study | | | |
| Intervention | | | | WASH | | | |
| Sample Group (N) | **Hookworm** | **T. trichiura** | **A. lumbricoides*** | | Infection intensity (**T. trichiura)** | Infection intensity (**Hookworm**) | Infection intensity (**A. lumbricoides**) |
| Baseline Control (372) | 13.7% (10.6–17.6)  1.1% (0.4–2.8) | 2.2% (1.1–4.3) | 48.7% (43.6–53.8) | | NA | NA | NA |
| Endline Control (303) | 9.9% (7.0–13.8) | 2.0% (0.8–4.3) | 23.4% (18.9–28.5) | | NA | NA | NA |
| Baseline Intervention (110) | 15.1% (9.4–23.3) | 1.9% (0.5–7.3) | 7.6% (3.8–14.4) | | NA | NA | NA |
| Endline Intervention (107) | 5.7% (2.5–12.1) | 0.9% (0.1–6.5%) | 0.9% (0.1–6.5) | | NA | NA | NA |
|  |  |  |  | |  |  |  |
|  | Stunting | Underweight | Thinness | | Anaemia |  |  |
| Baseline Control | 51.7% (46.6–56.8) N=382 | 53.3% (46.7–59.8)  N=225 | 25.5% (21.3–30.1)  N=382 | | 12.6% (9.6–16.3)  N=381 |  |  |
| Endline Control | 47.7% (42.3–53.2) N=356 | 58.4% (51.1–65.3) N=206 | 34.7% (29.7–40.1)  N=356 | | 4.9% (3.0–7.9) N=324 |  |  |
| Baseline Intervention | 62.1% (53.2–70.2) N=124 | 65.1% (54.4–74.5)  N=86 | 42.7% (34.3–51.6)  N=124 | | 4.3% (1.8–10.0)  N=124 |  |  |
| Endline Intervention | 66.1% (56.9–74.2) N=116 | 76.8% (66.4–84.8) N=83 | 47.8% (38.9–57.0)  N=116 | | 4.5% (1.9–10.3) N=112 |  |  |
| only a school-based WASH and deworming program (control arm); school-based + community-based WASH and deworming program in the community where the school was located (intervention arm). | | | | | | | |

| Eneanya, O. A., Gankpala, L., Goss, C. W., Bolay, F. K., Weil, G. J., & Fischer, P. U. (2021). Impact of Annual versus Semiannual Mass Drug Administration with Ivermectin and Albendazole on Helminth Infections in Southeastern Liberia. The American journal of tropical medicine and hygiene, 106(2), 700–709. https://doi.org/10.4269/ajtmh.21-0768 | |
| --- | --- |
| Data Extractors | Sommy Ugwu |
| Date of Data extraction | 18^th^ August, 2022 |
| Confirmation of Eligibility | Data on the effectiveness of CBI for STHs reported; baseline and endline data reported; specie specific data reported |
| Follow-up timeline | 4 years |
| Design | Before-after |
| Intervention | Albendazole + IVM |

| **Treatment zone** | **Number of subjects**  **(*N*)** | **Prevalence of *A. lumbricoides***  **(95% CI)** | **Arithmetic mean *epg* of *A. lumbricoides***  **(95% CI)** | **Prevalence Hookworm (95% CI)** | **Arithmetic mean *epg* of Hookworm**  **(95% CI)** | **Prevalence of *T. trichiura***  **(95% CI)** | **Arithmetic mean *epg* of**  ***T.s trichiura*** |
| --- | --- | --- | --- | --- | --- | --- | --- |
|  |  |  |  |  |  |  |  |
| **Annual** |  | 46.3 | 5754.9 | 23.6 | 125.0 | 36.5 | 200.3 |
| **MDA** (month 0) | 792 | (42.8, 49.9) | (4683.1, 6826.7) | (20.7, 26.7) | (87.2, 162.7) | (33.1, 40.0) | (166.1, 234.6) |
| Follow-up 1 (month 12) |  |  |  |  |  |  |  |
|  |  | 49.5 | 3016.5 | 8.5 | 37.7 | 8.6 | 25.5 |
|  | 729 | (45.8, 53.2) | (2340.5, 3692.5) | (6.5, 10.8) | (18.1, 57.4) | (6.7, 10.9) | (14.8, 36.1) |
| Follow-up 2  (month 24) |  |  |  |  |  |  |  |
|  |  | 57.9 | 3629.3 | 9.7 | 16.6 | 14.0 | 28.1 |
|  | 473 | (53.3, 62.4) | (2874.4, 4384.2) | (7.2, 12.8) | (10.4, 22.8 | (11.0, 17.4) | (12.8, 43.5) |
| Follow-up 3  (month 36) |  |  |  |  |  |  |  |
|  |  | 50.0 | 1566.9 | 8.8 | 12.3 | 7.4 | 5.9 |
|  | 444 | (45.2, 54.8) | (1171.8, 1962.0) | (6.3, 11.8) | (5.8, 18.7) | (5.2, 10.3) | (3.5, 8.4) |
| Follow-up 4  (month 72) |  |  |  |  |  |  |  |
|  |  | 55.2 | 1453.1 | 2.6 | 5.2 | 1.9 | 3.2 |
|  | 702 | (51.51, 59.0) | (1292.3, 1823.8) | (1.4, 3.9) | (2.8, 7.3) | (1, 3.1) | (1.6, 4.5) |
|  |  |  |  |  |  |  |  |
|  |  |  |  |  |  |  |  |
| **Semiannu** |  | 37.7 | 5302.6 | 28.7 | 214 | 38.4 | 285.0 |
| **al MDA**(month 0) | 698 | (34.1, 41.4) | (3966.2, 6639.0) | (25.3, 32.2) | (99.8, 328.3) | (34.8, 42.1) | (150.4, 419.7) |
| Follow-up 1  (month 12) |  |  |  |  |  |  |  |
|  |  | 34.1 | 2043.4 | 2.6 | 7.4 | 8.5 | 34.4 |
|  | 624 | (30.4, 38.0) | (1499.1, 2587.8) | (1.4, 4.1) | (1.6, 13.3) | (6.4, 11.0) | (-1.3, 70.0) |
| Follow-up 2  (month 24) |  |  |  |  |  |  |  |
|  |  | 41.2 | 700.5 | 13.2 | 26.6 | 11.3 | 38.7 |
|  | 425 | (36.5, 46.0) | (518.3, 882.7) | (10.1, 16.8) | (14.4, 38.8) | (8.4, 14.7) | (16.0, 61.4) |
| Follow-up 3  (month 36) |  |  |  |  |  |  |  |
|  |  | 40.8 | 1306.4 | 16.7 | 41.8 | 9.1 | 24.7 |
|  | 485 | (36.4, 45.3) | (912.3, 1700.4) | (13.5, 20.3) | (25.6, 58.0) | (6.7, 12.0) | (6.3, 43.0) |
| Follow-up 4  (month 72) |  |  |  |  |  |  |  |
|  |  | 66.8 | 1055.6 | 3.1 | 11.5 | 6.8 | 22.6 |
|  | 467 | (62.6, 71.3) | (879.3, 1298.3) | (1.8, 5.2) | (6.3, 16.4) | (4.6, 9.3) | (4.6, 38.3) |

| Bronzan, R. N., Dorkenoo, A. M., Agbo, Y. M., Halatoko, W., Layibo, Y., Adjeloh, P., Teko, M., Sossou, E., Yakpa, K., Tchalim, M., Datagni, G., Seim, A., & Sognikin, K. S. (2018). Impact of community-based integrated mass drug administration on schistosomiasis and soil-transmitted helminth prevalence in Togo. PLoS neglected tropical diseases, 12(8), e0006551. https://doi.org/10.1371/journal.pntd.0006551 | |
| --- | --- |
| Data Extractors | Sommy Ugwu |
| Date of Data extraction | 18^th^ August, 2022 |
| Confirmation of Eligibility | Data on the effectiveness of CBI for STHs reported; baseline and endline data reported; specie specific data reported |
| Follow-up timeline | 6 years |
| Design | Before-after |
| Intervention | Albendazole |

| **2009** | | | | | | | | **2015** | | | | | |
| --- | --- | --- | --- | --- | --- | --- | --- | --- | --- | --- | --- | --- | --- |
|  |  | **Hookworm** | | ***Ascaris*** | | ***Trichuris*** | | **Hookworm^c,g^** | | ***Ascaris*^d^** | | ***Trichuris*^d^** | |
|  |  | **N = 17097** | | **N = 17097** | | **N = 17097** | | **N = 16887** | | **N = 16888** | | **N = 16888** | |
| Prevalence of infection | | |  |  |  |  |  |  |  |  |  |  |  |
|  |  | n | (%) | N | (%) | n | (%) | N | (%) | n | (%) | n | (%) |
|  | | 5270 | (31.0) | 63 | (0.4) | 41 | (0.2) | 1868 | (11.1) | 52 | (0.3) | 62 | (0.4) |
| infected children with egg count data | | N = 5212 | | N = 63 | | N = 40 | | N = 1868 | | N = 52 | | N = 62 | |
|  |  | n (%) | | n (%) | | n (%) | | n (%) | | n (%) | | n (%) | |
|  | Heavy | 133 | (2.6) | 0 | (0.0) | 2 | (5.0) | 14 | (0.8) | 0 | (0.0) | 6 | (9.7) |
|  | Moderate | 164 | (3.2) | 10 | (15.9) | 7 | (17.5) | 38 | (2.0) | 9 | (17.3) | 14 | (22.6) |
|  | Light | 4915 | (94.3) | 53 | (84.1) | 31 | (77.5) | 1816 | (97.2) | 43 | (82.7) | 42 | (67.7) |
|  | Mean EPG^g^ | 570 | | 2358 | | 1682 | | 289 | | 2027 | | 3220 | |
|  | Median EPG | 168 | | 120 | | 72 | | 96 | | 144 | | 168 | |
|  | [range] | [24–36864] | | [24–26976] | | [24–19896] | | [24–9672] | | [24–16512] | | [24–66432] | |

| Lemos, M., Fançony, C., Moura, S., Mirante, C., Sousa, P., Barros, H., Nery, S., & Brito, M. (2020). Integrated community-based intervention for urinary schistosomiasis and soil-transmitted helminthiasis in children from Caxito, Angola. International health, 12(2), 86–94. https://doi.org/10.1093/inthealth/ihz055 | |
| --- | --- |
| Data Extractors | Sommy Ugwu |
| Date of Data extraction | 18^th^ August, 2022 |
| Confirmation of Eligibility | Data on the effectiveness of CBI for STHs reported; baseline and endline data reported; specie specific data reported |
| Follow-up timeline | 6 months |
| Design | Before-after |
| Intervention | praziquantel, albendazole and Coartem |

| **Indicators** | **STH (N=67)** | | |
| --- | --- | --- | --- |
|  | ***A. lumbricoides*** | ***T. trichiura*** | **Hookworms** |
| Baseline pretreatment (0) |  |  |  |
| No. of children infected | 4 | 6 | 2 |
| Prevalence: % (95% CI) | 6.0 (1.7 to 14.6) | 9.0 (3.4 to 18.5) | 3.0 (0.4 to 10.4) |
| Infection level: % (95% CI) |  |  |  |
| Light | 1.5 (0.0 to 8.0) | 9.0 (3.4 to 18.5) | 3.0 (0.4 to 10.4) |
| Moderate | 4.5 (0.9 to 12.5) | 0 | 0 |
| Heavy | 0 | 0 | 0 |
| GM eggs count (95% CI) | 4413 (11 to 13145) | 168 (60 to 366) | 55 (48 to 61) |
| 6 mo post-treatment (6) |  |  |  |
| No. of children infected | 3 | 2 | 0 |
| Prevalence: % (95% CI) | 4.5 (0.9 to 12.5) | 3.0 (0.4 to 10.4) | 0.0 (0.0 to 5.4) |
| Infection level: % (95% CI) |  |  |  |
| Light | 4.5 (0.9 to 12.5) | 1.5 (0.0 to 8.0) | 0 |
| Moderate | 0 | 1.5 (0.0 to 8.0) | 0 |
| Heavy | 0 | 0 | 0 |
| GM eggs count (95% CI) | 477 (24 to 1320) | 344 (192 to 509) | 0^3^ |
| PRR (0→6): % (p-value)¹ | 25.0 (1.000) | 66.7 (0.289) | 100 |
| ERR (0→6): % (p-value)^2^ | 89.2 (0.411) | −104.8 | - |
| Reinfection in % | 33.30 (1/3) | 0.0 (0/5) | 0.0 (0/2) |

| Pion, S. D. S., Chesnais, C. B., Weil, G. J., Louya, F., Boussinesq, M., & Missamou, F. (2021). Impact of Semi-Annual Albendazole on Lymphatic Filariasis and Soil-Transmitted Helminth Infection: Parasitological Assessment after 14 Rounds of Community Treatment. The American journal of tropical medicine and hygiene, 106(2), 729–731. https://doi.org/10.4269/ajtmh.21-0731 | | | | | | | |
| --- | --- | --- | --- | --- | --- | --- | --- |
| Data Extractors | | | | Sommy Ugwu | | | |
| Date of Data extraction | | | | 18^th^ August, 2022 | | | |
| Confirmation of Eligibility | | | | Data on the effectiveness of CBI for STHs reported; baseline and endline data reported; specie specific data reported | | | |
| Follow-up timeline | | | | 3 years | | | |
| Design | | | | Before-after | | | |
| Intervention | | | | albendazole | | | |
| Sample Group (N) | **Hookworm** | **T. trichiura** | **A. lumbricoides** | | Infection intensity (**T. trichiura)** | Infection intensity (**Hookworm**) | Infection intensity (**A. lumbricoides**) |
| Baseline (688) | 5% | 59.4% | 12.9% | | 356 | NA | 724 |
| Endline (383) | 0 | 42.9% | 13.6% | | 276 | NA | 589 |

| Gebrezgabiher, G., Yewhalaw, D., Ayana, M., Hailu, A., & Mekonnen, Z. (2022). Impact of ivermectin mass drug administration on burden of soil-transmitted helminths in onchocerciasis control and elimination programs, Yeki district, southwest Ethiopia. PloS one, 17(2), e0263625. https://doi.org/10.1371/journal.pone.0263625 | | | | | | | |
| --- | --- | --- | --- | --- | --- | --- | --- |
| Data Extractors | | | | Sommy Ugwu | | | |
| Date of Data extraction | | | | 18^th^ August, 2022 | | | |
| Confirmation of Eligibility | | | | Data on the effectiveness of CBI for STHs reported; baseline and endline data reported; specie specific data reported | | | |
| Follow-up timeline | | | | 10 years | | | |
| Design | | | | Before-after | | | |
| Intervention | | | | IVM | | | |
| Sample Group (N) | **Hookworm** | **T. trichiura** | **A. lumbricoides** | | Infection intensity (**T. trichiura)** | Infection intensity (**Hookworm**) | Infection intensity (**A. lumbricoides**) |
| Baseline (308) | 37.9% | 3.3% | 47.1% | | NA | NA | NA |
| Endline (400) | 15.5% | 27% | 10.8% | | NA | NA | NA |

| Hürlimann, E., Silué, K. D., Zouzou, F., Ouattara, M., Schmidlin, T., Yapi, R. B., Houngbedji, C. A., Dongo, K., Kouadio, B. A., Koné, S., Bonfoh, B., N'Goran, E. K., Utzinger, J., Acka-Douabélé, C. A., & Raso, G. (2018). Effect of an integrated intervention package of preventive chemotherapy, community-led total sanitation and health education on the prevalence of helminth and intestinal protozoa infections in Côte d'Ivoire. Parasites & vectors, 11(1), 115. https://doi.org/10.1186/s13071-018-2642-x | |
| --- | --- |
| Data Extractors | Sommy Ugwu |
| Date of Data extraction | 18^th^ August, 2022 |
| Confirmation of Eligibility | Data on the effectiveness of CBI for STHs reported; baseline and endline data reported; specie specific data reported |
| Follow-up timeline | 1 year |
| Design | Before-after |
| Intervention | preventive chemotherapy, community-led total sanitation (CLTS) and health education |

| **Parasite** | **Group** | **Baseline prevalence (%)** | **OR (95% CI)** | ***P*-value** | **Follow-up prevalence (%)** | **OR (95% CI)** | ***P*-value** | **Prevalence reduction (%)** |
| --- | --- | --- | --- | --- | --- | --- | --- | --- |
| Hookworm | Control | 25.19 | 1 |  | 7.01 | 1 |  | 18.18^*^ |
|  | Intervention | 36.47 | 1.70 (1.26–2.31) | 0.001 | 10.59 | 1.57 (0.95–2.58) | 0.076 | 25.88^*^ |
| *Ascaris lumbricoides* | Control | 0 | na |  | 0 | na |  | 0.00 |
|  | Intervention | 0.71 | na |  | 0.24 | na |  | 0.47 |
| *Trichuris trichiura* | Control | 1.30 | 1 |  | 0 | na |  | 1.30^*^ |
|  | Intervention | 2.59 | 2.02 (0.70–5.87) | 0.196 | 1.41 | na |  | 1.18 |

| Pion, S. D. S., Chesnais, C. B., Awaca-Uvon, N. P., Vlaminck, J., Abdou, A., Kunyu-Shako, B., Kuyangisa Simuna, G., Tambwe, J. P., Weil, G. J., & Boussinesq, M. (2020). The impact of four years of semiannual treatments with albendazole alone on lymphatic filariasis and soil-transmitted helminth infections: A community-based study in the Democratic Republic of the Congo. PLoS neglected tropical diseases, 14(6), e0008322. https://doi.org/10.1371/journal.pntd.0008322 | |
| --- | --- |
| Data Extractors | Sommy Ugwu |
| Date of Data extraction | 19^th^ August, 2022 |
| Confirmation of Eligibility | Data on the effectiveness of CBI for STHs reported; baseline and endline data reported; specie specific data reported |
| Follow-up timeline | 4 years |
| Design | Before-after community trial |
| Intervention | MDA with albendazole |

|  | **2014 (N = 413)** | **2015 (N = 214)** | **2016 (N = 308)** | **2017 (N = 252)** | **2018 (N = 184)** | **% reduction 2014–2018** | **p-value 2014–2018** |
| --- | --- | --- | --- | --- | --- | --- | --- |
| Rounds of mass drug administration before assessment | 0 | 2 | 4 | 6 | 8 |  |  |
| Hookworm |  |  |  |  |  |  |  |
| Number of positive samples | 242 | 98 | 118 | 103 | 39 |  |  |
| Prevalence (95% CI) | 58.6 (53.7–63.3) | 45.8 (39.2–52.5) | 38.3 (33.0–43.9) | 40.9 (34.9–47.1) | 21.2 (15.9–27.7) | 63.8% | < 0.0001 |
| Arithmetic mean eggs per g (95% CI) | 456.1 (319.2–593.0) | 251.0 (154.01–348.1) | 111 (66.7–155.3) | 66.9 (45.6–88.2) | 31.0 (14.8–47.1) | 93.2% | < 0.0001 |
| *Ascaris lumbricoides* |  |  |  |  |  |  |  |
| Number of positive samples | 58 | 5 | 7 | 9 | 3 |  |  |
| Prevalence (95% CI) | 14.0 (11.0–17.8) | 2.3 (1.0–5.5) | 2.3 (1.1–4.7) | 3.6 (1.9–6.7) | 1.6 (0.5–5.0) | 88.6% | < 0.0001 |
| Arithmetic mean eggs per g (95% CI) | 578.4 (305.1–851.6) | 173.0 (-84.5–430.5) | 176.5 (1.5–351.5) | 30.3 (-0.1–60.7) | 247.6 (-114.6–609.7) | 57.2% | 0.0858 |
| *Trichuris trichiura* |  |  |  |  |  |  |  |
| Number of positive samples | 17 | 5 | 11 | 12 | 5 |  |  |
| Prevalence (95% CI) | 4.1 (2.6–6.5) | 2.3 (1.0–5.5) | 3.6 (1.9–6.3) | 4.8 (2.7–8.2) | 2.7 (1.1–6.4) | 34.0% | 0.2011 |
| Arithmetic mean eggs per g (95% CI) | 8.0 (-0.8–17.0) | 10.5 (-4.9–25.9) | 31.3 (-11.7–74.3) | 28.4 (-12.9–69.6) | 3.4 (0.2–6.6) | 57.5% | 0.2499 |

| Loukouri, A., Méité, A., Koudou, B. G., Goss, C. W., Lew, D., Weil, G. J., N'Goran, E. K., & Fischer, P. U. (2020). Impact of annual and semi-annual mass drug administration for Lymphatic Filariasis and Onchocerciasis on Hookworm Infection in Côte d'Ivoire. *PLoS neglected tropical diseases*, *14*(9), e0008642. https://doi.org/10.1371/journal.pntd.0008642 | |
| --- | --- |
| Data Extractors | Sommy Ugwu |
| Date of Data extraction | 19^th^ August, 2022 |
| Confirmation of Eligibility | Data on the effectiveness of CBI for STHs reported; baseline and endline data reported; specie specific data reported |
| Follow-up timeline | 2 years |
| Design | Before-after community trial |
| Intervention | MDA with albendazole + IVM |

| **Hookworm** | **Baseline** | **1^st^ follow up** | **2^nd^ follow up** | **3^rd^ follow up** | **Reduction** |
| --- | --- | --- | --- | --- | --- |
| **Abengourou (1X)** |  |  |  |  |  |
| Prevalence % (95% CI) | 23.9^*^ (21.3–26.5) | 13.2 (11.2–15.2) | 7.3 (5.5–9.2) | 5.5^*^ (3.5–7.4) | 77 |
| Arithmetic mean epg (95% CI) | 406.2^**^ (306.3–506.2) | 260.4 (169.7–351.1) | 305.2 (81.2–529.3) | 118.3^**^ (77.9–158.8) | 70.9 |
| Level of intensity |  |  |  |  |  |
| No n (%) | 784 (76.12%) | 965 (86.78%) | 693 (92.65%) | 502 (94.54%) |  |
| **Akoupé (2X)** |  |  |  |  |  |
| Prevalence % (95% CI) | 12.4^*^ (10.3–14.4) | 7.5 (5.7–9.3) | 2.6 (1.4–3.8) | 1.9^*^ (0.8–3.0) | 85 |
| Arithmetic mean epg (95% CI) | 804.9^***^ (516.1–1093.7) | 135.0 (80.4–189.6) | 136.0 (82.7–189.3) | 875.0^***^ (0.0–2064.0) | −8.7 |
| Level of intensity |  |  |  |  |  |
| No n (%) | 869 (87.6%) | 739 (92.5%) | 665 (97.1%) | 618 (98.1%) |  |
| Total N (%) | 992 (100%) | 799 (100%) | 683 (100%) | 630 (100%) |  |

| Mwandawiro, C., Okoyo, C., Kihara, J., Simiyu, E., Kepha, S., Campbell, S. J., Freeman, M. C., Brooker, S. J., & Njenga, S. M. (2019). Results of a national school-based deworming programme on soil-transmitted helminths infections and schistosomiasis in Kenya: 2012-2017. Parasites & vectors, 12(1), 76. https://doi.org/10.1186/s13071-019-3322-1 | |
| --- | --- |
| Data Extractors | Sommy Ugwu |
| Date of Data extraction | 19^th^ August, 2022 |
| Confirmation of Eligibility | Data on the effectiveness of CBI for STHs reported; baseline and endline data reported; specie specific data reported |
| Follow-up timeline | 5 years |
| Design | Before-after |
| Intervention | MDA with albendazole or mebendazole |

|  | **Baseline** | **Midterm** | **Endline** | **Relative reduction (Baseline – Endline)** |
| --- | --- | --- | --- | --- |
| Survey prevalence, % (95% CI) | | | | |
| Hookworm | 15.4 (13.6–17.6) | 2.3 (1.8–3.0) | 1.3 (1.0–1.6) | 91.6 (*Z* = -21.34, *P* < 0.001) |
| *A. lumbricoides* | 18.1 (15.8–20.7) | 11.9 (10.2–13.9) | 9.6 (8.0–11.5) | 46.8 (*Z* = -10.82, *P* < 0.001) |
| *T. trichiura* | 6.7 (5.4–8.2) | 4.5 (3.4–6.0) | 4.1 (3.1–5.5) | 38.4 (*Z* = -5.38, *P* < 0.001) |
| Mean intensity, epg (95% CI) | | | | |
| Hookworm | 63 (50–81) | 8 (5–14) | 10 (5–19) | 84.2 (*Z* = -5.58, *P* < 0.001) |
| *A. lumbricoides* | 1659 (1378–1998) | 960 (801–1151) | 917 (750–1121) | 44.7 (*Z* = -8.27, *P* < 0.001) |
| *T. trichiura* | 33 (11–105) | 17 (11–26) | 16 (10–26) | 50.9 (*Z* = -1.15, *P* < 0.001) |

| Eneanya, O. A., Gankpala, L., Goss, C. W., Momolu, A. T., Nyan, E. S., Gray, E. B., Fischer, K., Curtis, K., Bolay, F. K., Weil, G. J., & Fischer, P. U. (2022). Community-based trial assessing the impact of annual versus semiannual mass drug administration with ivermectin plus albendazole and praziquantel on helminth infections in northwestern Liberia. Acta tropica, 231, 106437. https://doi.org/10.1016/j.actatropica.2022.106437 | |
| --- | --- |
| Data Extractors | Sommy Ugwu |
| Date of Data extraction | 19^th^ August, 2022 |
| Confirmation of Eligibility | Data on the effectiveness of CBI for STHs reported; baseline and endline data reported; specie specific data reported |
| Follow-up timeline | 4 years |
| Design | Before-after |
| Intervention | MDA with albendazole + IVM |

|  | **Ascaris** | | **Hookworm** | | **Trichuris** | |
| --- | --- | --- | --- | --- | --- | --- |
|  |  |  |  |  |  |  |
|  |  |  |  |  |  |  |
|  |  |  |  |  |  |  |
| **Treatment zone** | **Prevalence *(95% CI)*** | **Arithmetic mean *epg* (95% CI)** | **Prevalence (95% CI)** | **Arithmetic mean *epg* (95% CI)** | **Prevalence of (95% CI)** | **Arithmetic mean *epg* (95% CI)** |
|  |  |  |  |  |  |  |
| **Annual MDA** Baseline 762 | 2.2 (1.3, 3.5) | 0.7 (0.3, 1.1) | 47.5 (44.0, 51.1) | 182 (139.1, 224.9) | 0.5 (0.2, 1.4) | 20.4 (15.8, 56.6) |
|  |  |  |  |  |  |  |
| Follow-up 1 526 | 8.4 (6.1, 11.1) | 6.1 (3.0, 9.2) | 44.1 (39.8, 48.5) | 153.3 (107.1, 199.6) | 2.9 (1.6, 4.7) | 1 (0.3, 1.7) |
|  |  |  |  |  |  |  |
| Follow-up 2 720 | 16.3 (13.6, 19.2) | 7.5 (5.5, 9.5) | 29 (25.7, 32.4) | 33.2 (21.7, 44.6) | 4.7 (3.3, 6.5) | 14.0 (0.6, 28.6) |
|  |  |  |  |  |  |  |
| Follow-up 3 883 | 1.5 (0.8, 2.5) | 0.4 (0.1, 0.7) | 11 (9, 13.2) | 47.2 (28.3, 66.1) | 0.5 (0.1, 1.2) | 11.9 (8.4, 32.2) |
|  |  |  |  |  |  |  |
| Follow-up 4 1146 | 8.9 (7.1, 10.9) | 1.2 (0.5, 3.1) | 26 (23.2, 28.9) | 60.2 (48.2, 72.4) | 0.7 (0.3, 1.5) | 14.4 (10.3, 17.6) |
|  |  |  |  |  |  |  |
|  |  |  |  |  |  |  |
| **Semiannual MDA** Baseline1908 | 0.7 (0.4, 1.2) | 1.4 (0.8, 3.6) | 66.8 (64.6, 68.9) | 386.1 (350.3, 421.9) | 1.1 (0.7, 1.7) | 15.4 (2.2, 33.1) |
|  |  |  |  |  |  |  |
| Follow-up 1 1371 | 6.9 (5.6, 8.3) | 12.2 (4.5, 28.9) | 48.7 (46, 51.3) | 172.6 (143.7, 201.5) | 6.2 (5, 7.6) | 20.6 (2.7, 38.6) |
|  |  |  |  |  |  |  |
| Follow-up 2 1200 | 12.7 (10.8, 14.7) | 5.7 (4.3, 7.0) | 26.4 (23.9, 29) | 42.6 (25.9, 59.3) | 4.8 (3.7, 6.2) | 5.7 (0.2, 11.6) |
|  |  |  |  |  |  |  |
| Follow-up 3 1252 | 1.9 (1.2, 2.8) | 14.4 (11.7, 40.5) | 10.1 (8.5, 12) | 34.5 (23.9, 45) | 1.7 (1, 2.6) | 7.0 (1.4, 12.5) |
|  |  |  |  |  |  |  |
| Follow-up 4 1472 | 1.6 (1, 2.3) | 18.2 (12.9, 29.3) | 12.1 (10.5, 13.9) | 42.4 (33.1, 54.5) | 0.7 (0.3, 1.3) | 10.1 (6.2, 13.5) |
|  |  |  |  |  |  |  |
|  |  |  |  |  |  |  |

| Dyer, C. E., Ng-Nguyen, D., Clarke, N. E., Hii, S. F., Nguyen, H. Q., Nguyen, V. A. T., ... & Nery, S. V. (2023). Community-wide versus school-based targeted deworming for soil-transmitted helminth control in school-aged children in Vietnam: the CoDe-STH cluster-randomised controlled trial. The Lancet Regional Health–Western Pacific, 41. | | | | | | |
| --- | --- | --- | --- | --- | --- | --- |
| Data Extractors | | | Michael Muoka | | | |
| Date of Data extraction | | | 26^th^ January, 2024 | | | |
| Confirmation of Eligibility | | | Data on the effectiveness of CBI for Hookworm reported; baseline and endline data reported; specie specific data reported | | | |
| Follow-up timeline | | | 1 year | | | |
| Design | | | cRCT | | | |
| Intervention | | | MDA with albendazole | | | |
| BASELINE | | FOLLOW-UP | | | RELATIVE REDUCTION | |
| School **(N = 3797)** | Community **(N = 3838)** | School **(N = 3659)** | | Community **(N = 3717)** | School | Community |
| 16.7 (12.1–22.7) | 11.7 (7.8–17.3) | 11.7 (8.1–16.6) | | 7.7 (5.3–11.0) | 30.1 (20.5–39.6) | 34.6 (19.9–49.4) |

| Njenga, S. M., Mutungi, F. M., Wamae, C. N., Mwanje, M. T., Njiru, K. K., & Bockarie, M. J. (2014). Once a year school-based deworming with praziquantel and albendazole combination may not be adequate for control of urogenital schistosomiasis and hookworm infection in Matuga District, Kwale County, Kenya. Parasites & vectors, 7, 74. https://doi.org/10.1186/1756-3305-7-74 | |
| --- | --- |
| Data Extractors | Michael Muoka |
| Date of Data extraction | 26^th^ January, 2024 |
| Confirmation of Eligibility | Data on the effectiveness of CBI for STHs reported; baseline and endline data reported; specie specific data reported |
| Follow-up timeline | 3 years |
| Design | Before-after |
| Intervention | praziquantel and albendazole |

|  | **Sept 2009**  **N =**1022 | **Oct 2010**  **N=**1076 | **Mar 2011**  **N=**956 | **Jun 2012**  **N=**407 | **P-value** |  | |
| --- | --- | --- | --- | --- | --- | --- | --- |
|  | | | | | | |  |
| *A. lumbricoides** | \|  \| 12 (2.4%) \| \| --- \| --- \| | \|  \| 0 (0.0%) \| \| --- \| --- \| | \|  \| 0 (0.0%) \| \| --- \| --- \| | 1 (1.4%) |  | |  |
| Hookworm | 114 (22.6) | 14 (31.1%) | 16 (28.1%) | 7 (9.7%) |  | |  |
| *T. trichiura* | 10 (1.9%) | 0 (0.0%) | 1 (1.8%) | 1 (1.4%) |  | |  |
| **Arithmetric mean epg** **^a^(95% CI)** | | | | | | |  |
| *A. lumbricoides* | 21.5 (14.9-28) | 24 (2.7-45.3) | - | N/A | ND | |  |
| Hookworm | 269.5 (180.7-358.3) | 221 (176.9-265) | 277.2 (219.2-335.2) | 291.2 (204.7-377.6) | 0.5564 | |  |
| *T. trichiura* | 33.3 (24.1-42.4) | 31.2 (4.1-58.3) | 49.5 (-3.7-102.7) | 42 (4-80) | 0.3437 | |  |

| Nikolay B, Mwandawiro CS, Kihara JH, Okoyo C, Cano J, Mwanje MT, Sultani H, Alusala D, Turner HC, Teti C, Garn J, Freeman MC, Allen E, Anderson RM, Pullan RL, Njenga SM, Brooker SJ. Understanding Heterogeneity in the Impact of National Neglected Tropical Disease Control Programmes: Evidence from School-Based Deworming in Kenya. PLoS Negl Trop Dis. 2015 Sep 30;9(9):e0004108. doi: 10.1371/journal.pntd.0004108. PMID: 26421808; PMCID: PMC4589351. | |
| --- | --- |
| Data Extractors | Sommy Ugwu |
| Date of Data extraction | 19^th^ August, 2022 |
| Confirmation of Eligibility | Data on the effectiveness of CBI for STHs reported; baseline and endline data reported; specie specific data reported |
| Follow-up timeline | 2 years |
| Design | Before-after |
| Intervention | School-based MDA with albendazole |

| **Species** | **Baseline prevalence/ mean epg (95% CI)** | **Follow-up prevalence/ mean epg (95% CI)** | **Absolute reduction (%/ epg)** | **Relative reduction (%)** | **OR/epg ratio (95% CI)** | **p-value^1^** |
| --- | --- | --- | --- | --- | --- | --- |
| **Prevalence of infection** | | | | | | |
| STH combined | 34.76 (29.91; 40.39) | 19.68 (15.28; 25.34) | 15.08 | 43.38 | 0.44 (0.42; 0.47) | <0.001 |
| *A*. *lumbricoides* | 23.17 (18.19; 29.52) | 15.44 (12.00; 19.86) | 7.73 | 33.36 | 0.59 (0.56; 0.62) | <0.001 |
| Hookworm | 14.61 (9.84; 21.69) | 1.75 (0.85; 3.60) | 12.86 | 88.02 | 0.10 (0.08; 0.11) | <0.001 |
| *T*. *trichiura* | 6.28 (3.29; 12.01) | 5.38 (2.51; 11.51) | 0.9 | 14.33 | 0.83 (0.75; 0.91) | <0.001 |
| **Average intensity of infection** | | | | | | |
| *A*. *lumbricoides* | 2,147 (1,420; 3,246) | 1,248 (929; 1,675) | 899 | 41.89 | 0.62 (0.47; 0.83) | 0.002 |
| Hookworm | 63 (34; 118) | 7 (2; 21) | 56 | 88.92 | 0.06 (0.03; 0.10) | <0.001 |
| *T*. *trichiura* | 40 (11; 140) | 21 (8; 54) | 19 | 48.32 | 0.78 (0.46; 1.33) | 0.364 |

| Pullan RL, Halliday KE, Oswald WE, Mcharo C, Beaumont E, Kepha S, Witek-McManus S, Gichuki PM, Allen E, Drake T, Pitt C, Matendechero SH, Gwayi-Chore MC, Anderson RM, Njenga SM, Brooker SJ, Mwandawiro CS. Effects, equity, and cost of school-based and community-wide treatment strategies for soil-transmitted helminths in Kenya: a cluster-randomised controlled trial. Lancet. 2019 May 18;393(10185):2039-2050. doi: 10.1016/S0140-6736(18)32591-1. Epub 2019 Apr 18. PMID: 31006575; PMCID: PMC6525786. | |
| --- | --- |
| Data Extractors | Sommy Ugwu |
| Date of Data extraction | 19^th^ August, 2022 |
| Confirmation of Eligibility | Data on the effectiveness of CBI for STHs reported; baseline and endline data reported; specie specific data reported |
| Follow-up timeline | 2 years |
| Design | Before-after |
| Intervention | School-based MDA with albendazole |

|  |  | **School-based deworming** | **Annual community-wide treatment** | **Biannual community-wide treatment** |
| --- | --- | --- | --- | --- |
| **Participants with soil-transmitted helminth data (n=19 684)** | | | | |
| Total included | | 6396 | 6523 | 6765 |
| Prevalence of soil-transmitted helminth infection | | | | |
|  | Hookworm | 1187 (18·6%) | 1168 (17·9%) | 1396 (20·6%) |
|  | *Ascaris lumbricoides* | 30 (0·5%) | 18 (0·3%) | 30 (0·4%) |
|  | *Trichuris trichiura* | 272 (4·3%) | 189 (2·9%) | 250 (3·7%) |
| Mean intensity of soil-transmitted helminth infection (eggs per gram) | | | | |
|  | Hookworm | 169·7 (1248·2) | 175·1 (1965·2) | 158·2 (1002·3) |
|  | *Ascaris lumbricoides* | 62·6 (2023·3) | 18·9 (648·2) | 31·0 (808·7) |
|  | *Trichuris trichiura* | 12·7 (205·0) | 8·3 (145·0) | 29·3 (1245·8) |

|  |  | **Number positive of total respondents** | **Community prevalence (95% CI)** | **Absolute percentage change from baseline (95% CI)** | **Unadjusted risk ratio (95% CI)** | **p value** | **Adjusted risk ratio^*^(95% CI)** | **p value** |
| --- | --- | --- | --- | --- | --- | --- | --- | --- |
| **Hookworm** | | | | | | | | |
| 12-month assessment | | | | | | | | |
|  | School-based deworming | 1284 of 7957 | 16·1% (12·1 to 20·1) | −2·4% (−10·9 to 6·1) | 1 (ref) | .. | 1 (ref) | .. |
|  | Annual community-wide treatment | 984 of 8355 | 11·8% (9·0 to 14·6) | −6·1% (−13·6 to −1·3) | 0·73 (0·52 to 1·03) | .. | 0·77 (0·65 to 0·91) | .. |
|  | Biannual community-wide treatment | 836 of 8177 | 10·2% (7·6 to 12·9) | −10·4% (−15·5 to −6·0) | 0·64 (0·45 to 0·92) | 0·04 | 0·65 (0·53 to 0·78) | <0·001 |
| 24-month assessment | | | | | | | | |
|  | School-based deworming | 972 of 7058 | 13·8% (10·5 to 17·0) | −4·8% (−13·0 to 3·5) | 1 (ref) | .. | 1 (ref) | .. |
|  | Annual community-wide treatment | 597 of 7446 | 8·0% (6·0 to 10·1) | −9·9% (−16·8 to −3·0) | 0·59 (0·42 to 0·83) | .. | 0·64 (0·52 to 0·78) | .. |
|  | Biannual community-wide treatment | 453 of 7281 | 6·2% (4·9 to 7·5) | −14·4% (−21·4 to −7·4) | 0·46 (0·33 to 0·63) | <0·001 | 0·48 (0·41 to 0·57) | <0·001 |
| ***Trichuris trichiura*** | | | | | | | | |
| 12-month assessment | | | | | | | | |
|  | School-based deworming | 296 of 7957 | 3·7% (1·8 to 5·7) | −0·5% (−6·2 to 5·2) | 1 (ref) | .. | 1 (ref) | .. |
|  | Annual community-wide treatment | 223 of 8355 | 2·7% (1·7 to 3·6) | −0·2% (−4·5 to 4·1) | 0·70 (0·37 to 1·32) | .. | 1·18 (0·80 to 1·74) | .. |
|  | Biannual community-wide treatment | 287 of 8177 | 3·5% (1·9 to 5·2) | −0·2% (−5·6 to 5·2) | 0·90 (0·45 to 1·81) | 0·47 | 1·16 (0·82 to 1·65) | 0·63 |
| 24-month assessment | | | | | | | | |
|  | School-based deworming | 292 of 7058 | 4·1% (1·9 to 6·4) | −0·1% (−5·9 to 5·7) | 1 (ref) | .. | 1 (ref) | .. |
|  | Annual community-wide treatment | 197 of 7446 | 2·6% (1·7 to 3·6) | −0·3% (−4·7 to 4·2) | 0·65 (0·34 to 1·24) | .. | 1·20 (0·86 to 1·68) | .. |
|  | Biannual community-wide treatment | 237 of 7281 | 3·3% (1·8 to 4·7) | −0·4% (−5·8 to 4·9) | 0·80 (0·40 to 1·61) | 0·41 | 1·01 (0·77 to 1·34) | 0·43 |

**Table B. QUALITY ASSESSMENT SUMMARY TABLE**

| Study | D1 | D1b | D2 | D3 | D4 | D5 | D6 | D7 | Overall |
| --- | --- | --- | --- | --- | --- | --- | --- | --- | --- |
| Freeman et al. 2013 | Low | Low | Some concerns | Low | some concerns | Low | NA | NA | Some concerns |
| Gyorkos et al. 2013 | Low | Low | Low | Low | Low | Low | NA | NA | Low |
| Al-delaimy et al. 2014 | Low | some concerns | some concerns | Low | Low | Low | NA | NA | some concerns |
| Al-Mekhalfi et al. 2014 | Low | Low | low | Low | Low | Low | NA | NA | Low |
| Njenga et al. 2014 | Moderate | Low | Low | serious | Serious | Low | Low | Low | Serious |
| Nikolay et al. 2015 | Low | NA | Low | Low | Low | Low | Low | Low | Low |
| Pion et al. 2015 | Moderate | NA | Low | Low | Moderate | Moderate | Low | Low | Moderate |
| Sunish et al. 2015 | Low | NA | Low | Low | Moderate | Moderate | Low | Low | Moderate |
| Okoyo et al. 2017 | Low | NA | Low | Low | Moderate | Moderate | Low | Low | Moderate |
| Ash et al. 2017 | Low | NA | Low | Low | Low | Moderate | Low | Low | Moderate |
| Echazu et al. 2017 | Low | NA | Low | Low | Low | Moderate | Low | Low | Moderate |
| Paige et al. 2017 | High | Some concerns | some concerns | Some concerns | Low | Low | NA | NA | Some concerns |
| Pion et al. 2017 | Moderate | NA | Low | Low | Moderate | Moderate | Low | Low | Moderate |
| Clarke et al. 2018 | Low | NA | Low | Low | Low | Moderate | Low | Low | Moderate |
| Bronzan et al. 2018 | Serious | NA | Serious | Low | Moderate | Moderate | Low | Low | Serious |
| Hürlimann et al. 2018 | Low | NA | Low | Low | Moderate | Moderate | Low | Low | Serious |
| Dunn et al. 2019 | Low | NA | Low | Low | Moderate | Moderate | Low | Low | Moderate |
| Lemos et al. 2019 | Low | NA | Low | Low | Serious | Serious | Low | Low | Serious |
| Mwandawiro et al. 2019 | Low | NA | Low | Low | Moderate | Moderate | Low | Low | Moderate |
| Vas Nery et al. 2019 | Low | some concerns | some concerns | Low | Low | Low | NA | NA | some concerns |
| Pullan et al. 2019 | Low | Low | Low | Low | Low | Low | NA | NA | Low |
| Loukouri et al. 2020 | Low | NA | Low | Low | Low | Moderate | Low | Low | Moderate |
| Pion et al. 2020 | Moderate | NA | Low | Low | Serious | Moderate | Low | Low | Serious |
| Chen et al. 2021 | Low | NA | Low | Low | Low | Moderate | Low | Low | Moderate |
| Eneanya et al. 2021 | Moderate | NA | Low | Low | Serious | Serious | Moderate | Low | Serious |
| Eneanya et al. 2022 | Moderate | NA | Low | Low | Moderate | Serious | Low | Low | Serious |
| Gebrezgabiher et al. 2022 | Moderate | NA | Low | Critical | Critical | Low | Serious | Low | Serious |
| Landeryou et al. 2022 | Low | NA | Low | Low | Moderate | Moderate | Low | Low | Moderate |
| Muslim & Lim 2022 | Low | NA | Low | Low | Moderate | Serious | Low | Low | Serious |
| Pion et al. 2022 | Serious | NA | Low | Low | Moderate | Moderate | Low | Low | Serious |
| Dyer et al. 2023 | Low | Low | Low | Low | Low | Low | NA | NA | Low |
| Le et al. 2023a | Low | Low | Low | Low | Low | Low | NA | NA | Low |
| Le et al. 2023b | Low | NA | Low | Low | Low | Low | Low | Low | Low |

**Table C. FULL SEARCH TERMS USED**

**Global Health Online (Ovid), Embase (Ovid)**

| “community based interventions” | Communit* OR “primary health*” OR “health* worker*” OR “outreach” OR “public participat*” OR “patient participat*” |
| --- | --- |
| Soil-transmitted Helminths | ascari* OR roundworm* OR schistosom* OR bilharz* OR “blood-fluke” OR helminth* OR STH* OR trichuri* OR whipworm* OR “soil transmit*” OR trematod* OR hookworm* OR Ancylostoma*OR Necator* OR “neglected tropical disease*” OR “NTD*” |

**MEDLINE (Ovid), Cochrane Library, Web of Science**

| “community based interventions” | Communit* OR “primary health*” OR “health* worker*” OR “outreach” OR “public participat*” OR “patient participat*” |
| --- | --- |
| Soil-transmitted Helminths | ascari* OR roundworm* OR bilharz* OR “blood-fluke” OR helminth* OR STH* OR trichuri* OR whipworm* OR “soil transmit*” OR trematod* OR hookworm* OR Ancylostoma*OR Necator* OR “neglected tropical disease*” OR “NTD*” |
