# Supplementary material for: The impact of community based interventions for the prevention and control of soil-transmitted helminths: A systematic review and meta-analysis
Source: PLOS Glob Public Health. 2024 Oct 10;4(10):e0003717. doi: 10.1371/journal.pgph.0003717 (PMC11466416; doi:10.1371/journal.pgph.0003717)
Supplement: Attachment — Submitted filename: PGPH-D-23-01261_reviewer.pdf [file pgph.0003717.s005.pdf]

PLOS Global Public Health

The Impact of Community Based Interventions for the Prevention and Control of Soil-Transmitted Helminths in sub-Saharan Africa: A Systematic Review and Meta-analysis

--Manuscript Draft--

**Manuscript Number:**	PGPH-D-23-01261
**Article Type:**	Research Article
**Full Title:**	The Impact of Community Based Interventions for the Prevention and Control of Soil-Transmitted Helminths in sub-Saharan Africa: A Systematic Review and Meta-analysis
**Short Title:**	The Impact of CBI for the Prevention and Control of STH in sub-Saharan Africa
**Corresponding Author:**	Sommy Ugwu<br>LSHTM: London School of Hygiene & Tropical Medicine<br>London, UNITED KINGDOM
**Order of Authors:**	Sommy Ugwu
	Michael Obinna Muoka
	Clara MacLeod
	Sarah Bick
	Oliver Cumming
	Laura Braun
**Keywords:**	soil-transmitted helminths; Community-based interventions; Neglected tropical diseases; Mass drug administration; Hygiene
**Abstract:**	Background: Soil-transmitted helminths (STHs) are among the most common human infections worldwide and a major cause of morbidity. They are caused by different species of parasitic worms and transmitted by eggs released in faeces. The main control strategy in endemic regions is periodic treatment with deworming medication. In the last 10 years, there has been a scale-up of prevention and control activities with a focus on community-based interventions (CBIs). This review aims to systematically analyse the impact of CBIs on the prevalence and infection of STH infection in sub-Saharan Africa. A systematic review was published on this topic in 2014, but there have subsequently been several new studies published which are included in this updated review.Method: Electronic database search of MEDLINE (Ovid), Global Health Online (Ovid), Cochrane Library, Embase (Ovid) and Web of Science was conducted. Titles, abstracts, and full texts were screened by two independent reviewers according to predefined eligibility criteria. Data were extracted and a meta-analysis of included studies was conducted.Results: A total of 2329 de-duplicated titles were screened, and 18 studies were included in the review. 13 focussed on community-wide treatment while seven studies investigated school-based interventions, and two studies investigating both. Results suggest that CBIs are effective in reducing the prevalence of Hookworm (Relative risk [RR]: 0.24, 95% CI: 0.17, 0.34), Trichuris trichiura (RR: 0.74, 95% CI: 0.57, 0.95) and Ascaris lumbricoides (RR: 0.70, 95% CI 0.55, 0.88). School-based treatment and community-wide treatment, as well as annual and semi-annual deworming, all reduce STH prevalence significantly.<br>Conclusions: Results suggest that CBIs are effective in reducing the prevalence and intensity of STH infections. While most studies delivered preventive chemotherapy (PC), few studies explored the impact of interventions such as water, sanitation, and hygiene (WASH) or health education, which may be essential in preventing reinfection after PC.
**Additional Information:**	
**Question**	**Response**
**Financial Disclosure**	The authors received no financial support for the research, authorship or publication of this article.

Powered by Editorial Manager® and ProduXion Manager® from Aries Systems Corporation

Enter a financial disclosure statement that describes the sources of funding for the work included in this submission and the role the funder(s) played. This includes grants and any commercial funding of the work or authors.<br><br>This statement will be typeset if the manuscript is accepted for publication.<br><br>*Please review the submission guidelines and the instructions link below for detailed requirements and guidance.*	
**Competing Interests**<br><br>On behalf of all authors, disclose any competing interests that could be perceived to bias this work.<br><br>This statement will be typeset if the manuscript is accepted for publication.<br><br>*Please review the instructions link below and PLOS Global Public Health's competing interests policy to determine what information must be disclosed at submission.*	We declare no competing interest.
**Data Availability**<br><br>Before publication, Authors are required to make fully available and without restriction all data underlying their findings. Please see our PLOS Data Policy page for detailed information on this policy.<br><br>A **Data Availability Statement**, detailing where the data can be accessed, is required at first submission. Insert your Data Availability Statement in the box below.	All data reported in the manuscript are provided as part of the submitted article or in the supplementary documentation.

Please see the data reporting section of our submission guidelines for instructions on what you need to include in your Data Availability Statement.<br><br>This statement will be typeset if the manuscript is accepted for publication.<br><br>*PLOS allows rare exemptions to address legal and ethical concerns. If you have legal or ethical restrictions, please use the box below to detail these in full sentences for the Journal team to consider.*	

Manuscript Click here to access/download;Manuscript;CBI_STH.docx

The Impact of Community Based Interventions for the Prevention and Control of Soil-Transmitted Helminths in sub-Saharan Africa: A Systematic Review and Meta-analysis

Sommy C Ugwu[1*], Michael O Muoka[2], Clara MacLeod[1], Sarah Bick[1], Oliver Cumming[1], Laura Braun[1]

1 London School of Hygiene and Tropical Medicine, London, United Kingdom
2 Zenith Medical and Kidney Centre, Nigeria
*Corresponding author

Abstract

Background: Soil-transmitted helminths (STHs) are among the most common human infections worldwide and a major cause of morbidity. They are caused by different species of parasitic worms and transmitted by eggs released in faeces. The main control strategy in endemic regions is periodic treatment with deworming medication. In the last 10 years, there has been a scale-up of prevention and control activities with a focus on community-based interventions (CBIs). This review aims to systematically analyse the impact of CBIs on the prevalence and infection of STH infection in sub-Saharan Africa. A systematic review was published on this topic in 2014, but there have subsequently been several new studies published which are included in this updated review.

Method: Electronic database search of MEDLINE (Ovid), Global Health Online (Ovid), Cochrane Library, Embase (Ovid) and Web of Science was conducted. Titles, abstracts, and full texts were screened by two independent reviewers according to predefined eligibility criteria. Data were extracted and a meta-analysis of included studies was conducted.

Results: A total of 2329 de-duplicated titles were screened, and 18 studies were included in the review. 13 focussed on community-wide treatment while seven studies investigated school-based interventions, and two studies investigating both. Results suggest that CBIs are effective in reducing the prevalence of Hookworm (Relative risk [RR]: 0.24, 95% CI: 0.17, 0.34), *Trichuris trichiura* (RR: 0.74, 95% CI: 0.57, 0.95) and *Ascaris lumbricoides* (RR: 0.70, 95% CI 0.55, 0.88). School-based treatment and community-wide treatment, as well as annual and semi-annual deworming, all reduce STH prevalence significantly.

Conclusions: Results suggest that CBIs are effective in reducing the prevalence and intensity of STH infections. While most studies delivered preventive chemotherapy (PC), few studies explored the impact of interventions such as water, sanitation, and hygiene (WASH) or health education, which may be essential in preventing reinfection after PC.

Introduction

Soil-transmitted helminths (STHs) are a sub-group of neglected tropical diseases (NTDs) that continue to be transmitted in Africa, the Americas, and East Asia [1]. STHs include roundworms (*Ascaris lumbricoides*), whipworms (*Trichuris trichiura*) and hookworms (*Ancylostoma duodenale* and *Necator americanus*) [2]. They represent a vast public health problem, with approximately 1.5 billion people infected worldwide [3]. Over 70% of the mortality burden lies in sub-Saharan Africa [3]. Infection occurs through ingestion of embryonated eggs or when larvae in the soil penetrate the skin [4].

Children, pregnant women, and young adults living in low- and middle-income countries (LMICs) are disproportionately affected by STHs and experience more severe sequelae [5]. The worldwide burden of STH infection was estimated to be responsible for up to 39 million disability-adjusted life years (DALYs), resulting from diarrhoea, anaemia, malnutrition, physical and mental growth retardation and other adverse health outcomes [1, 6]. These infections are perpetuated by, and reinforce social determinants of poverty, illiteracy, poor

nutrition, and a lack of access to health education as well as safe water, sanitation and hygiene (WASH) [7].

The World Health Organization's (WHO) 2021–2030 road map for NTDs targets the elimination of STHs as a public health problem by 2030 (defined as <2% of moderate-to-heavy intensity infection) [7]. Regular preventive chemotherapy (PC) with the anthelminthic drugs albendazole or mebendazole is recommended by the WHO in areas where prevalence exceeds 20%. This can control morbidity, but reinfection is likely to continue if populations continue to live in highly contaminated environments due to inadequate WASH infrastructure. Interventions including health education and WASH are therefore also recommended [7]. Progress in delivering PC has been in part driven by the donations of billions of anthelmintic drugs from the pharmaceutical industry [8], which contribute to the cost-effectiveness of control programs. Drugs are often delivered through schools or the community, which provides an effective platform for drug administration.

A community-based intervention (CBI) includes any intervention delivered in a community setting, defined here as domestic, public and institutional settings, rather than traditional hospital-based care delivered in health facilities. In reference to STHs, this can include interventions such as health education, WASH, distribution of shoes and PC. Delivery mode of PC includes community-wide treatment (CWT) and school-based treatment (SBT). The advantage of SBT is that it makes use of existing infrastructure to target school-aged children, the age group most at risk of STH infection. Nonetheless, CWT may be important for interrupting the transmission of STHs since infected adults or pre-school aged children not reached through SBT remain infe[illegible]. WHO guidelines recommend annual PC for school-aged children in areas where ST[illegible]evalence in children is between 20%-50% and semi-annual PC in communities where it exceeds 50% [9].

CBIs can be integrated or non-integrated. Integrated CBIs are delivered as part of a pre-existing routine prevention and control program. Interventions ca[illegible] integrated with WASH or drug treatment targeting other diseases. Integrated WASH inte[illegible]ions refer to those that augment PC with WASH while integrated chemotherapy for NDTs refers to a combination of separate PC programs targeting multiple NTDs including schistosomiasis, lymphatic filariasis, onchocerciasis, and trachoma.

A previous systematic review conducted in 2014 found that CBIs were effective in reducing the prevalence and infection intensity of STHs [10]. The review also found that CBIs reduced the prevalence of anaemia but had no impact on anthropometric indices. There is a need to update these findings, especially considering the scaling up of prevention a[illegible]ontrol programs in the last decade as well as evidence of reduced drug efficacy in areas with high drug pressure [11].

Aims

The aim of this review is to critically evaluate the effectiveness of CBIs for the prevention and control of STHs in Sub-Saharan Africa. A previous review on the same topic was published in 2014 with literature [illegible]ch conducted in 2013 [10]. This study seeks to update the findings of the previous review.

Methods

Search strategy

Search terms relating to the three concepts of this review, namely "Community bas[illegible] interventions", "Helminth disease" and "sub-Saharan Africa", were included. The search ter[illegible] were refined following a test search. The full list of search terms with synonyms is provided in the Supplementary Data. Electronic database searches were conducted in MEDLINE (Ovid), Global Health Online (Ovid), Cochrane Library, Embase (Ovid) and Web of Science. Search

terms were mapped to MeSH terms in MEDLINE, Cochrane and Embase libraries. The search field was limited to title and abstract. Searches were conducted on 30 July 2022 and were restricted to articles published from 2013, which is when the search of the previously published review was conducted [10]. Additionally, reference lists of included studies were screened manually to identify relevant studies. The review protocol was not registered.

Eligibility criteria

Peer-reviewed [illegible]les published after April 2013 were eligible for incl[illegible]n if they reported on the effectiveness of the community-based delivery of interventions f[illegible]THs. To focus this review, only studies conducted in sub-Saharan Africa were included. Randomised, quasi-randomised and before-and-after studies were included in the meta-analysis in which intervention was carried out in the community and reported outcomes (prevalence or intensity of infection) could be compared to baseline measures or a control group, and reported per species, not combined. Non-peer reviewed articles or grey literature were excluded. Interventions that were delivered in the hospital setting were not eligible for inclusion since they are not considered CBIs. Cross-sectional studies, case reports, conference abstracts, qualitative studies and secondary research articles were also excluded. Studies were not excluded based on language of publication.

Study screening and selection

Following database search, all results were exported to Mendeley reference manager software and de-duplicated. Two reviewers (SU, CM) independently screened titles and abstracts for eligibility. The full texts of remaining studies were sought and screened by the two reviewers. Any unsolved discrepancies were discussed with the third reviewer (LB). Reasons for exclusion of studies were documented at each step.

Quality appraisal of included studies

An adapted version of the Cochrane risk of bias tool (RoB2) [12] was used for quality appraisal of randomised trials, while the risk of bias in non-randomized studies - of Interventions tool (ROBINS-I) [13] was adapted and used to appraise non-randomised studies. The included studies were categorised as having very poor, poor, fair or good quality. Two co-authors (SU and MM) independently assessed the quality, and any discrepancies were resolved with a third author (LB).

Outcome measures

The primary outcomes a[illegible]STH prevalence (reported per species) and intensity of infection (eggs per gram of sto[illegible]econdary outcomes are anaemia, stunting and any reported nutritional outcomes and growth indices.

Data extraction

A data extraction table was created in Excel based on the data collection form available from The Cochrane Collaboration [14]. Study outcome data and characteristics, including study aims, design, methods, setting, sample size, participant characteristics, time frame, type of intervention, limitations and funding sources, were systematically extracted for each study.

Data synthesis and analysis

Following data extraction, a meta-analysis was conducted using comparable outcome data from included studies in Review Manager version 5.4 software [15]. Only studies reporting primary outcomes infection for at least one STH species are included in the meta-analysis. For randomised trials and controlled before-and-after studies, endline intervention and control outcome data are compared. For uncontrolled studies, baseline and endline are compared. Where multiple follow-ups were conducted, the data from the last follow-up was extracted.

Effect measures were reported as relative risk (RR) for prevalence and standard mean difference (SMD) for infection intensity with 95% confidence intervals (CI). The arithmetic mean of eggs per gram of stool (epg) was used as a measure for intensity of infection. This is calculated using data from all participants, unlike the geometric mean only includes data from positive egg counts (>0 epg). If not provided, standard deviations for infection intensity were calculated using the sample size and confidence intervals provided, as per the Cochrane Handbook [14].

Sub-group analyses were conducted on treatment frequency (annual vs semi-annual) as well treatment delivery (school-based vs community-based). Analyses were run in Review Manager, pooling and comparing the relative risk of sub-groups. For studies that could not be included in the meta-analysis, a narrative synthesis is conducted.

Results

The electronic search of five databases resulted in a total of 5,070 records. No additional studies were found from citation follow-up and search of reference list of included studies. All records were imported into Mendeley reference manager where 2741 duplicates were removed. The titles and abstracts of the remaining articles were screened according [illegible] eligibility criteria including full text review of 52 articles. In total, 18 articles met the inclusi[illegible] criteria and were included in this synthesis. The PRISMA flowchart presents further details.

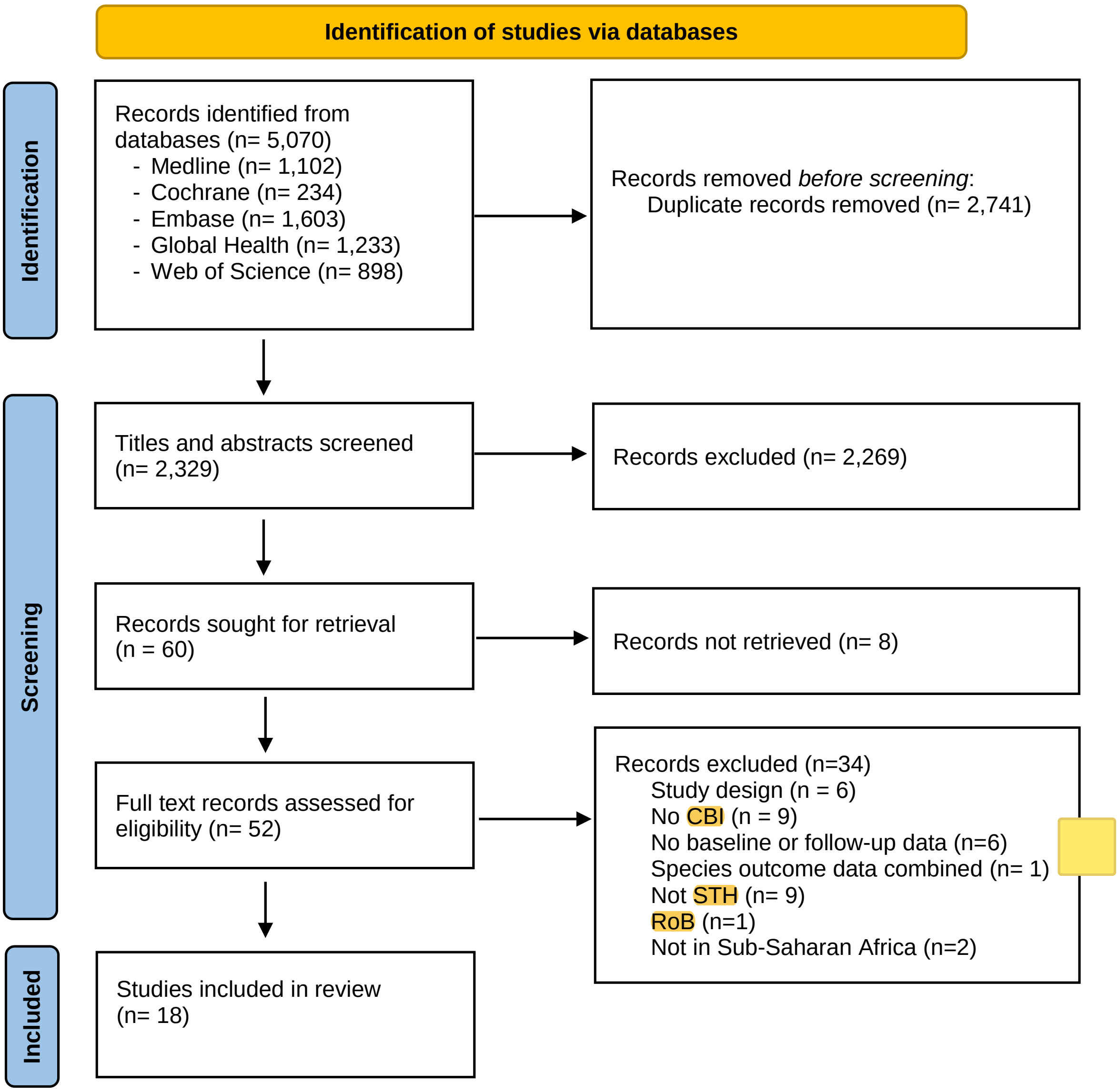

Figure 1: PRISMA diagram

Characteristics of included studies

18 studies are included in this review. [illegible] of the included studies focused on PC delivering mebendazole or albendazole as the pr[illegible]y CBI (15/18). Seven studies investigated school-based interventions while 13 focussed community-based drug treatments. Only three studies investigate WASH, and one investigates shoe wearing. The majority of included studies were conducted in Kenya (28%), followed by the Democratic Republic of Congo (22%), Côte d'Ivoire (17%) and Liberia (11%). One study was each conducted in Uganda, Angola, Ethiopia and Togo. All studies reported on hookworm, whereas two studies did not report on *A. lumbricoides* or *T. trichiura*. Most studies (16/18) used a before-and-after study design and two were cluster randomised controlled trials (cRCT). Key characteristics of included studies are presented in Table 1

Table 1: Characteristics of included studies

**Author**	**Country**	**Study Setting**	**Study Design**	**Study Population**	**Species and outcome**	**Type of Intervention**	**Quality**
Freeman et al. 2013 [16]	Kenya	Nyanza Province, western Kenya	Cluster RCT	N=913<br>7 - 13 years old	STH	School-based WASH	Poor
Njenga et al. 2014 [17]	Kenya	Matuga District, Kwale County	Before-after study	N=1022<br>7-12 years old	STH	School-based	PC
Nikolay et al. 2015 [18]	Kenya	schools surveyed in western Kenya	Before-after study	N=21,528 school-aged children	STH	SBT	Good
Pion et al. 2015 [19]	Democratic republic of Congo	Seke Pembe	Before-after study	N= 773<br>≥ 5 years old	STH	CWT	Poor
Okoyo et al. 2016 [20]	Kenya	Western region, Rift Valley, Coast and Nyanza.	Before-after study	N= 21,432<br>2-9 years old	STH	SBT	Poor
Paige et al. 2017 [21]	Uganda	Kabarole District, western Uganda	Controlled before-after study	245<br>>4 years old	STH	Provision of footwear with public health iconography	Poor
Pion et al. 2017 [22]	Democratic republic of Congo	Seke Pembe	Before-after study	N= 462<br>≥5 years or older	STH	CWT	Fair
Bronzan et al. 2018 [23]	Togo	Binah district	Before-after	N= 17,100 children no age range	STH	Integrated CWT	Very poor
Hürlimann et al. 2018 [24]	Côte d'Ivoire	South-central Côte d'Ivoire	Controlled before-after study	N= 1894 no age range	STH	CWT + Sanitation and health education	Fair
Lemos et al. 2019 [25]	Angola	Cabungo	Before-after study	N= 209<br>2-15 years old	STH<br>Anaemia	Integrated WASH and CWT	Poor
Mwandawiro et al. 2019 [26]	Kenya	across several regions in Kenya	Before-after study	21,528<br>2-24 years old	STH	SBT	Fair
Pullan et al. 2019 [27]	Kenya	Kwale County	Cluster RCT	N= 19,684<br>≥ 2 years old	STH	SBT and CWT	Good
Loukouri et al. 2020 [28]	Côte d'Ivoire	Forest zone of eastern Côte d'Ivoire	Before-after study	2,022 no age range	Hookworm	CWT	Fair
Pion et al. 2021 [29]	Democratic republic of Congo	Mbunkimi and Misay	Before-after study	N=1,266 All<br>≥ 5 years old	STH	CWT	Fair

Eneanya et al. 2021 [30]	Liberia	Maryland County, southeastern Liberia	Before-after study	N= 7,838<br>≥ 5 years old	STH	CWT	Poor
Eneanya et al 2022 [31]	Liberia	Lofa County, northern Liberia	Before-after study	N=3464<br>≥ 5 years old	STH	CWT	Fair
Gebrezgabiher et al. 2022 [32]	Ethiopia	Yeki district, SNNPR, southwest Ethiopia	Before-after	N= 308<br>≥10 years old	STH	CWT	Poor
Pion et al. 2022 [33]	Democratic republic of Congo	Seke Pembe	Before-after study	N= 383<br>11-14 years old	STH	CWT	Poor

Quality assessment of included studies

Two studies were judged to have good quality, seven as fair, eight as poor and one as very poor. The study with very poor quality used convenience sampling to select participants and had issues with respecting the maximum slide reading time. The completed risk of bias table is available in the supplementary data.

Effect of CBIs on STH prevalence and intensity

14 studies are included in the meta-analysis. Out of these, 13 reported on Hookworm prevalence, and 12 each on *A. lumbricoides* and *T. trichiura* prevalence. Pooled analyses show that following CBI, the prevalence is reduced by 76% for hookworm (RR: 0.24, 95% CI: 0.17, 0.34), 30% for *A. lumbricoides* (RR: 0.70, 95% CI 0.55, 0.88) and 26% for *T trichiura* (RR: 0.74, 95% CI: 0.57, 0.95) (Figure 2). The total pooled effect estimate is 0.48 (95% CI: 0.39, 0.58) for all STHs combined. Although several studies had wide effect margins, most found that CBIs reduced the prevalence of STHs. No studies reported a significant increase in prevalence for hookworm, though two studies from Liberia reported a statistically significant increase in *A. Lumbricoides* prevalence following CBI [30, 31]. Authors suggest this may be caused by a higher re-infection of *A. Lumbricoides* compared to other helminths [30]. This may also be linked to the disruption in PC due to the Ebola outbreak. For *T. Trichiura,* the study from Togo reported a significant increase in prevalence following five years of annual PC (RR: 21.31, 95% CI: 15.59, 29.13) [23]. The authors noted that *T. Trichiura* infection was very rare, likely due to a long history of onchocerciasis-focussed ivermectin distribution. 97% of STH infections were accounted for by Hookworm infections [23]. The study had serious risk of bias as the slide reading time foreseen in the protocol was not always respected, and convenience sampling was used. Paige and colleagues assessed the efficacy of a sandal (*Holoflop*) with educational imagery attached in a community receiving regular school-based deworming [21]. They found no significant reduction in STH prevalence.

Effect of CBIs on intensity of infection

This meta-analysis indicates that CBI are also effective in reducing infection intensity for STHs overall (SMD -109.46, 95% CI: -135.65, -83.27), as well as for all species (Figure 3). The largest reduction in intensity was found for Hookworm (SMD -132.24, 95% CI: -179.34, -85.14), followed by *A. lumbricoides* (SMD -120.17, 95% CI: -190.38, -49.96). The mean values of *A. lumbricoides* intensity were significantly higher than those reported for the other two species of STH, indicating higher infection rates. Most endline intensities reported were of low or medium classification.

Few studies reported differing effects of CBIs on prevalence and intensity. Eneanya et al found prevalence of *A. lumbricoides* to increase following annual [30, 31] and semi-annual [30] community-wide PC, yet the intervention was associated with decreases in infection intensities (geometric mean epg) ($P < 0.001$ in both annual and semi-annual treatment) [30]. All other studies were consistent or did not report both prevalence and intensity.

Study or Subgroup	Weight	Risk Ratio M-H, Random, 95% CI
**1.1.1 Hookworm**		
Bronzan et al. 2018	2.3%	0.36 [0.34, 0.38]
Eneanya et al. 2021 (1)	2.0%	0.11 [0.07, 0.17]
Eneanya et al. 2021 (2)	2.0%	0.10 [0.06, 0.18]
Eneanya et al. 2022 (3)	2.3%	0.55 [0.48, 0.62]
Eneanya et al. 2022 ((4)	2.3%	0.18 [0.16, 0.21]
Lemos et al. 2019	1.0%	0.59 [0.13, 2.62]
Loukouri et al. 2020 (5)	2.1%	0.23 [0.16, 0.33]
Loukouri et al. 2020 (6)	1.9%	0.15 [0.09, 0.28]
Mwandawiro et al. 2019	2.3%	0.08 [0.07, 0.09]
Nikolay et al. 2015	2.3%	0.11 [0.10, 0.13]
Okoyo et al. 2016	2.3%	0.15 [0.14, 0.16]
Paige et al. 2017 (7)	1.2%	0.84 [0.26, 2.77]
Paige et al. 2017 (8)	1.5%	1.19 [0.47, 3.03]
Pion et al. 2015	1.0%	0.09 [0.02, 0.38]
Pion et al. 2017	0.4%	0.02 [0.00, 0.35]
Pion et al. 2020	2.2%	0.36 [0.27, 0.48]
Pion et al. 2022	0.4%	0.02 [0.00, 0.32]
Pullan et al. 2019 (9)	2.3%	0.45 [0.41, 0.49]
Pullan et al. 2019 (10)	2.3%	0.30 [0.27, 0.33]
Pullan et al. 2019 (11)	2.3%	0.74 [0.69, 0.80]
**Subtotal (95% CI)**	**36.2%**	**0.24 [0.17, 0.34]**
Total events		
Heterogeneity: $Tau^2 = 0.49$; $Chi^2 = 1870.54$, df = 19 ($P < 0.00001$); $I^2 = 99\%$		
Test for overall effect: Z = 8.18 ($P < 0.00001$)		
**1.1.2 *A. lumbricoides***		
Bronzan et al. 2018	2.1%	0.84 [0.58, 1.21]
Eneanya et al. 2021 (12)	2.3%	1.19 [1.08, 1.32]
Eneanya et al. 2021 (13)	2.3%	1.77 [1.58, 1.99]
Eneanya et al. 2022 (14)	2.0%	3.99 [2.41, 6.61]
Eneanya et al. 2022 ((15)	2.2%	0.85 [0.66, 1.10]
Lemos et al. 2019	1.4%	0.44 [0.16, 1.20]
Mwandawiro et al. 2019	2.3%	0.53 [0.51, 0.56]
Nikolay et al. 2015	2.3%	0.86 [0.82, 0.90]
Okoyo et al. 2016	2.3%	0.66 [0.63, 0.69]
Paige et al. 2017 (16)	1.4%	1.27 [0.45, 3.60]
Paige et al. 2017 (17)	0.6%	0.21 [0.03, 1.72]
Pion et al. 2015	2.3%	0.58 [0.49, 0.68]
Pion et al. 2017	2.2%	0.20 [0.15, 0.27]
Pion et al. 2020	1.3%	0.12 [0.04, 0.37]
Pion et al. 2022	2.2%	0.24 [0.18, 0.31]
**Subtotal (95% CI)**	**29.0%**	**0.70 [0.55, 0.88]**
Total events		
Heterogeneity: $Tau^2 = 0.16$; $Chi^2 = 758.83$, df = 14 ($P < 0.00001$); $I^2 = 98\%$		
Test for overall effect: Z = 3.04 (P = 0.002)		
**1.1.3 *T. trichiura***		
Bronzan et al. 2018	2.2%	21.31 [15.59, 29.13]
Eneanya et al. 2021 (18)	1.9%	0.05 [0.03, 0.09]
Eneanya et al. 2021 (19)	2.1%	0.18 [0.13, 0.25]
Eneanya et al. 2022 (20)	1.2%	1.33 [0.40, 4.40]
Eneanya et al. 2022 ((21)	1.7%	0.62 [0.29, 1.31]
Lemos et al. 2019	1.6%	0.61 [0.26, 1.40]
Mwandawiro et al. 2019	2.3%	0.62 [0.57, 0.67]
Nikolay et al. 2015	2.3%	0.81 [0.75, 0.88]
Okoyo et al. 2016	2.3%	0.67 [0.62, 0.73]
Paige et al. 2017 (22)	1.7%	0.54 [0.25, 1.13]
Paige et al. 2017 (23)	0.4%	1.04 [0.07, 16.19]
Pion et al. 2015	2.3%	0.90 [0.83, 0.98]
Pion et al. 2017	2.3%	0.76 [0.69, 0.83]
Pion et al. 2020	1.4%	0.66 [0.25, 1.76]
Pion et al. 2022	2.3%	0.54 [0.48, 0.62]
Pullan et al. 2019 (24)	2.3%	0.97 [0.83, 1.14]
Pullan et al. 2019 (25)	2.2%	0.91 [0.75, 1.11]
Pullan et al. 2019 (26)	2.3%	0.95 [0.80, 1.12]
**Subtotal (95% CI)**	**34.8%**	**0.74 [0.57, 0.95]**
Total events		
Heterogeneity: $Tau^2 = 0.24$; $Chi^2 = 718.82$, df = 17 ($P < 0.00001$); $I^2 = 98\%$		
Test for overall effect: Z = 2.33 (P = 0.02)		
**Total (95% CI)**	**100.0%**	**0.48 [0.39, 0.58]**
Total events		
Heterogeneity: $Tau^2 = 0.43$; $Chi^2 = 5720.12$, df = 52 ($P < 0.00001$); $I^2 = 99\%$		
Test for overall effect: Z = 7.43 ([illegible]		
Test for subgroup differences: C [illegible] = 93.6%		

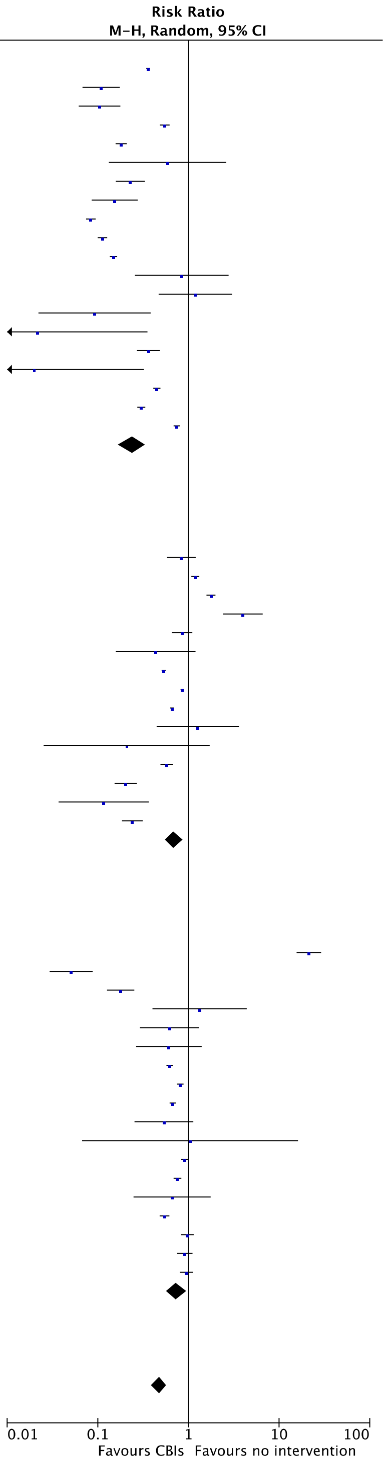

Footnotes

(1) Annual MDA
(2) Semi-annual MDA
(3) Annual
(4) Semi-annual
(5) Annual MDA
(6) Semi-annual MDA
(7) Holoflop
(8) Normal shoes
(9) Annual MDA
(10) Semi-annual MDA
(11) SBD
(12) Annual MDA
(13) Semi-annual MDA
(14) Annual
(15) Semi-annual
(16) Holoflop
(17) normal shoes
(18) Annual MDA
(19) Semi-annual MDA
(20) Annual
(21) Semi-annual
(22) Holoflop
(23) Normal shoes
(24) SBD
(25) Annual MDA
(26) Semi-annual MDA

Figure 2: Forest plot showing the impact of CBIs on the prevalence of STHs

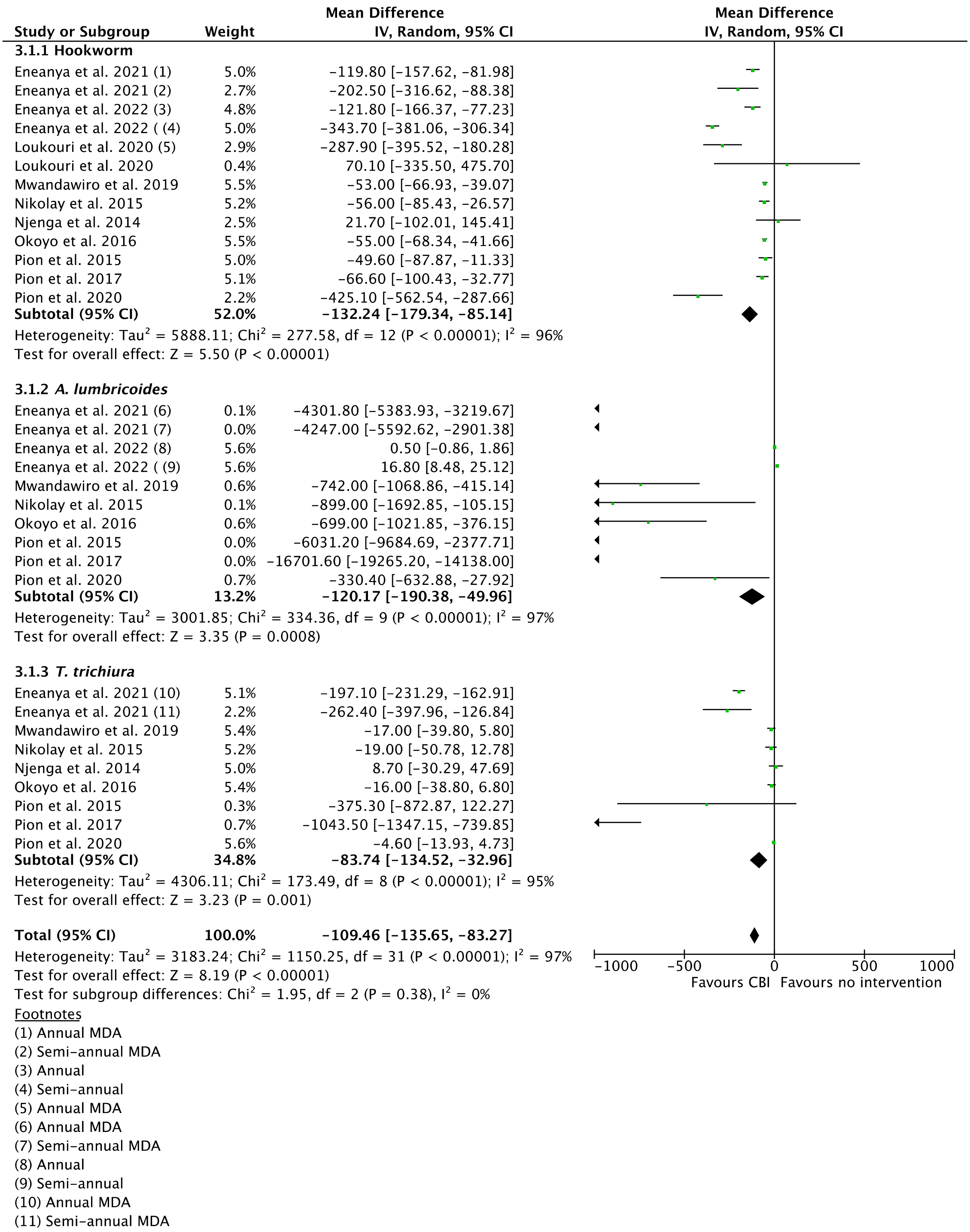

Figure 3: Forest plot of the impact of CBIs on STH infection intensity

Three studies are excluded from the meta-analysis as enrolment numbers per treatment arm or baseline/endline are not provided [16, 24, 32]. This includes a cluster-RCT in Kenya assessing the impact of school-based WASH that only found a significant effect on *A. lumbricoides* prevalence and intensity, but not on *T. trichiura* or hookworm [16]. A study from Côte d'Ivoire that implemented PC alongside community-led total sanitation (CLTS) and health education found [illegible]dence of the intervention significantly decreasing STH infection [24]. Gebrezgabiher evaluated the impact of long-term CWT on STH infection with the drug

ivermectin, primarily used to treat onchocerciasis. Prevalence of all STH infection significantly decreased from baseline to endline over the course of this multi-year study [32].

Semi-annual versus annual mass drug administration
A subgroup analysis was conducted to compare semi-annual vs annual PC for each STH species as well as for all STH species combined (table 2). The number of studies included in annual subgroup analysis varied between five to eight, with a total of 20 studie[illegible]aluating semi-annual PC and 19 studies annual PC. Pooled analysis for STHs shows no difference in prevalence reduction between semi-annual (0.38 [0.28, 0.53]) and annual treatment (0.53 [0.41, 0.69]) frequency. This also holds for individual species. Although species-specific prevalence reductions differed for treatment frequencies, this was not significant.

Table 2: effect estimates (relative risk and 95% confidence interval) for semi-annual and annual PC

	Semi-annual PC		Annual PC	
	Datasets included	Risk ratio	Datasets included	Risk ratio
Hookworm	8	0.25 [0.20, 0.32] I^2 86%	7	0.16 [0.09, 0.28], I^2 99%
A. lumbricoides	5	0.38 [0.14, 1.02], I^2 99%	6	0.75 [0.59, 0.96], I^2 98%
T. trichiura	7	0.68 [0.51, 0.92], I^2 96%	6	0.53 [0.41, 0.69], I^2 96%
STH combined	20	0.38 [0.28, 0.53], I^2 99%	19	0.37 [0.27, 0.51], I^2 96%

Comparison of community wide treatment and school-based deworming
Seven studies delivered interventions in school settings, treating school children only, and 13 delivered community-wide treatment (table 3). Both [illegible]hool-based and community-wide treatment are found to be effective in reducing ST[illegible]revalence. However, there is no significant difference between treatment delivery of PC. The biggest reduction in prevalence for both delivery platforms was found for hookworm (75% reduction for CWT and 83% reduction for SBT), followed by *A. lumbricoides* and *T. trichiura.* The subgroup analysis for CWT revealed a pooled STH relative risk of 0.38 (95% CI 0.29, 0.49) for CWT and 0.43 (95% CI 0.31, 0.61] for SBT. There were limited data to compare integrated versus non-integrated approaches.

Table 3: effect estimates (relative risk and 95% confidence interval) for CWT and SBT

	CWT		SBT	
	Datasets included	Risk ratio	Datasets included	Risk ratio
Hookworm	8	0.25 [0.21, 0.31], I^2 90%	7	0.17 [0.06, 0.48], I^2 100%
A. lumbricoides	5	0.51 [0.28, 0.91], I^2 98%	6	0.65 [0.51, 0.84], I^2 98%
T. trichiura	7	0.55 [0.39, 0.77], I^2 97%	6	0.72 [0.61, 0.84], I^2 88%
STH combined	20	0.38 [0.29, 0.49], I^2 99%	19	0.43 [0.31, 0.61], I^2 100%

Secondary outcomes of interest were only reported in only study in Angola, which reported reduction in the prevalence of anaemia from 55.2% to 40.9% among children who received CWT [25]. The integrated intervention, treating STHs, malaria and schistosomiasis, found strong association between anaemia and urinary schistosomiasis (*S. haematobium*) and no *A. lumbricoides* infections were found in severe cases.

There are too few data to determine the impact of health education on STH infection. One included study reported a significant difference in prevalence reduction for Hookworm among those who received community-led total sanitation and health education compared to those who received PC alone (Difference in proportions = -0.10 95% CI: -0.16, -0.04) [24].

Discussion

This meta-analysis suggests that CBIs are effective in reducing both the intensity and prevalence of STH infection. Not all studies reported both the prevalence and intensity of infection, and even fewer reported prevalence of intensity categories (i.e., categories based on epg). These additional data help explore the effect of CBIs on STH infection, as changes in intensity categories may be overlooked if only assessing the mean intensity of infection.

The pooled risk ratio for the impact of CBIs on hookworm infection (RR: 0.24, 95% CI: 0.17, 0.33) was the lowest, indicating greater benefit of the intervention compared to that for *A. lumbricoides* and *T. trichiura.* One included study reported a longer time-to-reading of Kato-Katz slides [illegible] foreseen in the protocol, exceeding the time threshold for detecting ho[illegible]orm eggs which are prone to degradation [34]. The study only reported a significant inte[illegible]tion effect for hookworm infection, not *A. lumbricoides* or *T. trichiura*. Though other authors did not report similar egg counting issues, it may have reduced Kato-Katz sensitivity for hookworm detection, especially as time-to-reading was not reported in many studies.

The difference in pooled effect for Hookworm compared to *A. lumbricoides* and *T. trichiura* may indicate that re-infection occurs more quickly for *Ascaris and Trichuris.* The differences can also be attributed to differences in drug efficacy by dose, helminth species, individuals' age, or infection intensity. Anthelmintic drugs may also be more effective for Hookworm compared to the other STHs in sub-Saharan [illegible]a. This is supported by evidence from a recent study showing that from 2000 - 2018 the prevalence of hookworm fell significantly from 30% to 5%, with less significant reductions for *A. lumbricoides* (17% to 9%) and *T. trichiura* (12% to 2%) [35].

Many studies included in the review delivered integrated control programmes, including simultaneous treatment of STHs, schistosomiasis and lymphatic filariasis. Integrated programs can reduce the burden of multiple diseases and reach many people at risk [36]. However, there are limited data to compare integrated versus non-integrated approaches in this review.

Diagnostics

Studies used the Kato-Katz method [37] for STH detection, which is recommended by the WHO. However, this diagnostic method is less sensitive than other methods, such as PCR, especially in regions with low infection intensity and prevalence. This is because only a small sample of stool is analysed per slide (41.7 mg) and eggs are not evenly dispersed in stool. Therefore, egg counts and prevalence at low infection intensities may be under-estimated [38]. More specific and sensitive diagnostic tools suitable for low-resource settings are required to enable a test-and-treat approach, and to help determine when PC can be stopped.

The number of slides analysed varied between studies. Most analysed the recommended duplicate stool samples, though some analysed only a single slide per participant [23]. In addition, the number of smears sometimes varied between baseline and follow-up measures, affecting primary outcome measures.

The results of the meta-analysis had an I^2 value ranging from 95-100% for STH indicating a high level of heterogeneity. A high level of heterogeneity was expected for this meta-analysis for several reasons. First, CBI is a broad term that includes interventions such as PC, sanitation, health education and provision of shoes. Additionally, these interventions can be delivered at different intervals including monthly, bi-annually and annually. The included studies also have varying risk of bias with assessed quality ranging from very poor to good (see supplementary table). This assumption is validated by the lower I^2 observed during subgroup analysis. Other factors that can contribute to heterogeneity include variations in study population, study duration, research method and healthcare systems [14]. A meta-

regression could be conducted to explore which study characteristics explain variation between studies, such as follow-up time, intervention, publication date.

The findings are in agreement with those of the previous review conducted [10]. Very similar reductions in STH prevalence were found by Salam et al (reduction of 55% (RR: 0.45, 95% CI: 0.38, 0.54) versus a reduction of 53% here. Trichuriasis prevalence displayed the lowest reduction following CBI, 34% in Salam and 28% in this review. Hookworm and Ascariasis prevalence was reduced by 60% (95% CI: 0.31, 0.52) and 68% (95% CI: 0.20, 0.51) respectively, whereas by 76% and 32% in this review. The smaller effect on *A. lumbricoides* prevalence in this review is partially due to the two studies from Liberia which contributed four datasets in total and reported low treatment compliance (39%-56%).

Limitations

This review was focussed to sub-Saharan Africa and is therefore not generalisable to other regions. STHs disproportionately affect children, and sub-group analyses could be conducted to determine effects on age groups. Results from the previous review coul[illegible] pooled with the results of this review to ensure sufficient datasets for analysis. The scope of the review could be broadened to include data on outcomes such re-infection intensity, cognitive development, and behavioural outcomes.

Several studies had multiple follow-up timepoints for measuring prevalence and intensity of infection, and endline data are not all from the same follow-up time. It was not possible to extract data for the same follow-up time as study designs and lengths differed. This may explain differences in prevalence and infection intensity between studies. Future reviews could conduct sub-group analyses to explore the impact of follow-up time on prevalence and intensity of infection.

The analysis calculates effect sizes and standard errors using raw data extracted from studies with different designs. This can introduce bias, as raw data from cluster trials are unadjusted for clustering, and the standard errors may be underestimated. Si[illegible]ly, non-randomised studies may adjust for covariates to reduce confounding, which will not be captured in the analysis. Some studies only provided effects instead of raw data and were therefore not included in the meta-analysis. Future reviews could extract effect sizes, if provided, and make conversions.

Mean egg counts before and after intervention were used to assess the effect of CBIs. To avoid normalising the data variance and excluding very high intensity infections, the arithmetic mean of infection intensity was extracted instead of the geometric mean. However, this may not be the best way to assess intervention effects [39]. In addition to changes in arithmetic means, changes in infection intensity categories (e.g., very high to high) should be assessed using statistical tests.

Conclusions

This review examined the impact of CBIs on STH prevalence and infection in sub-Saharan Africa. Community delivery platforms are increasingly being advocated for the prevention and control of many public health issues, and this review suggests that they can be effective in reducing prevalence and infection intensity of STHs. Most CBIs focussed on PC, which reduces the prevalence of STH and can prevent severe disease. However, it cannot prevent reinfection in the long term which is likely if environments continue to be contaminated due to open defecation or unimproved sanitation facilities. With the added concerns of drug resistance in areas with high drug pressure, it is key for research to focus on sustainable interventions that prevent reinfection.

The importance of comprehensive sanitation, hygiene and health education is not reflected in the number of published studies. These interventions play a role in preventing reinfection and

should be integrated into existing programs for maximum benefit. Therefore, further research is required to develop more effective strategies if the target of eliminating STHs by 2030 is to be achieved.

List of abbreviations

CBI Community-based intervention
CWT Community-wide treatment
NTD neglected tropical disease
PC Preventive Chemotherapy
SBD School-based deworming
STH Soil-transmitted helminths
WASH Water, Sanitation and Hygiene

Declarations

Ethics approval and consent to participate: Not applicable

Consent for publication: Not applicable

Availability of data and materials: All data generated during this study are included in this published article and its supplementary information files.

Competing interests: The authors declare that they have no competing interests" in this section.

Funding: None

Authors' contributions: LB and SU developed the protocol for this review. LB, SU and CM screened papers. MM assessed quality of studies and SB advises on statistical analyses. LB and SU conducted analyses and all authors contributed to data interpretation and manuscript writing.

Acknowledgements: We would like to thank the LSHTM librarians for their support in developing the search strategy and finding full-text papers.

References

1. Collaborators, *Global, regional, and national incidence, prevalence, and years lived with disability for 354 diseases and injuries for 195 countries and territories, 1990–2017: a systematic analysis for the Global Burden of Disease Study 2017.* The Lancet, 2018. **392**(10159): p. 1789-1858.
2. Bethony, J., et al., *Soil-transmitted helminth infections: ascariasis, trichuriasis, and hookworm.* Lancet, 2006. **367**(9521): p. 1521-32.
3. World Health Organization, *Gobal Health Estimates 2020: Disease burden by Cause, Age, Sex, by Country and by Region, 2000-2019.* . 2020: Geneva.
4. Jourdan, P.M., et al., *Soil-transmitted helminth infections.* Lancet, 2018. **391**(10117): p. 252-265.

5. World Health Organization, *Soil-transmitted helminth infections: factsheet*. 2023: Geneva.
6. Bhutta, Z.A., et al., *Global burden, distribution, and interventions for infectious diseases of poverty.* Infect Dis Poverty, 2014. **3**: p. 21.
7. World Health Organization, *Ending the neglect to attain the Sustainable Development Goals: A road map for neglected tropical diseases 2021–2030*, M.M. Ntuli, Editor. 2021: Geneva.
8. Montresor, A., et al., *Reduction in DALYs lost due to soil-transmitted helminthiases and schistosomiasis from 2000 to 2019 is parallel to the increase in coverage of the global control programmes.* PLOS Neglected Tropical Diseases, 2022. **16**(7): p. e0010575.
9. World Health Organization, *Guideline: preventive chemotherapy to control soil-transmitted helminth infections in at-risk population groups*. 2017: Geneva.
10. Salam, R.A., et al., *Community-based interventions for the prevention and control of helmintic neglected tropical diseases.* Infectious Diseases of Poverty, 2014. **3**(1): p. 23.
11. Walker, M., et al., *Individual responses to a single oral dose of albendazole indicate reduced efficacy against soil-transmitted helminths in an area with high drug pressure.* PLOS Neglected Tropical Diseases, 2021. **15**(10): p. e0009888.
12. Sterne, J.A.C., et al., *RoB 2: a revised tool for assessing risk of bias in randomised trials.* BMJ, 2019. **366**: p. l4898.
13. Sterne, J.A.C., et al., *ROBINS-I: a tool for assessing risk of bias in non-randomised studies of interventions.* BMJ, 2016. **355**: p. i4919.
14. JPT, H., et al., *Cochrane Handbook for Systematic Reviews of Interventions version 6.3 (updated February 2022).* 2022: Cochrane.
15. The Cochrane Collaboration. *Review Manager (RevMan)*. 2020.
16. Freeman, M.C., et al., *The impact of a school-based hygiene, water quality and sanitation intervention on soil-transmitted helminth reinfection: a cluster-randomized trial.* Am J Trop Med Hyg, 2013. **89**(5): p. 875-83.
17. Njenga, S.M., et al., *Once a year school-based deworming with praziquantel and albendazole combination may not be adequate for control of urogenital schistosomiasis and hookworm infection in Matuga District, Kwale County, Kenya.* Parasit Vectors, 2014. **7**: p. 74.
18. Nikolay, B., et al., *Understanding Heterogeneity in the Impact of National Neglected Tropical Disease Control Programmes: Evidence from School-Based Deworming in Kenya.* PLOS Neglected Tropical Diseases, 2015. **9**(9): p. e0004108.
19. Pion, S.D., et al., *The impact of two semiannual treatments with albendazole alone on lymphatic filariasis and soil-transmitted helminth infections: a community-based study in the Republic of Congo.* Am J Trop Med Hyg, 2015. **92**(5): p. 959-66.
20. Okoyo, C., et al., *Monitoring the impact of a national school based deworming programme on soil-transmitted helminths in Kenya: the first three years, 2012 – 2014.* Parasites & Vectors, 2016. **9**(1): p. 408.
21. Paige, S.B., et al., *Combining Footwear with Public Health Iconography to Prevent Soil-Transmitted Helminth Infections.* Am J Trop Med Hyg, 2017. **96**(1): p. 205-213.
22. Pion, S.D.S., et al., *Effect of 3 years of biannual mass drug administration with albendazole on lymphatic filariasis and soil-transmitted helminth infections: a*

community-based study in Republic of the Congo. The Lancet Infectious Diseases, 2017. **17**(7): p. 763-769.
23. Bronzan, R.N., et al., *Impact of community-based integrated mass drug administration on schistosomiasis and soil-transmitted helminth prevalence in Togo.* PLOS Neglected Tropical Diseases, 2018. **12**(8): p. e0006551.
24. Hürlimann, E., et al., *Effect of an integrated intervention package of preventive chemotherapy, community-led total sanitation and health education on the prevalence of helminth and intestinal protozoa infections in Côte d'Ivoire.* Parasites & Vectors, 2018. **11**(1): p. 115.
25. Lemos, M., et al., *Integrated community-based intervention for urinary schistosomiasis and soil-transmitted helminthiasis in children from Caxito, Angola.* International Health, 2020. **12**(2): p. 86-94.
26. Mwandawiro, C., et al., *Results of a national school-based deworming programme on soil-transmitted helminths infections and schistosomiasis in Kenya: 2012-2017.* Parasit Vectors, 2019. **12**(1): p. 76.
27. Pullan, R.L., et al., *Effects, equity, and cost of school-based and community-wide treatment strategies for soil-transmitted helminths in Kenya: a cluster-randomised controlled trial.* The Lancet, 2019. **393**(10185): p. 2039-2050.
28. Loukouri, A., et al., *Impact of annual and semi-annual mass drug administration for Lymphatic Filariasis and Onchocerciasis on Hookworm Infection in Côte d'Ivoire.* PLOS Neglected Tropical Diseases, 2020. **14**(9): p. e0008642.
29. Pion, S.D.S., et al., *The impact of four years of semiannual treatments with albendazole alone on lymphatic filariasis and soil-transmitted helminth infections: A community-based study in the Democratic Republic of the Congo.* PLoS Negl Trop Dis, 2020. **14**(6): p. e0008322.
30. Eneanya, O.A., et al., *Impact of Annual versus Semiannual Mass Drug Administration with Ivermectin and Albendazole on Helminth Infections in Southeastern Liberia.* Am J Trop Med Hyg, 2021. **106**(2): p. 700-709.
31. Eneanya, O.A., et al., *Community-based trial assessing the impact of annual versus semiannual mass drug administration with ivermectin plus albendazole and praziquantel on helminth infections in northwestern Liberia.* Acta Trop, 2022. **231**: p. 106437.
32. Gebrezgabiher, G., et al., *Impact of ivermectin mass drug administration on burden of soil-transmitted helminths in onchocerciasis control and elimination programs, Yeki district, southwest Ethiopia.* PLOS ONE, 2022. **17**(2): p. e0263625.
33. Pion, S.D.S., et al., *Impact of Semi-Annual Albendazole on Lymphatic Filariasis and Soil-Transmitted Helminth Infection: Parasitological Assessment after 14 Rounds of Community Treatment.* Am J Trop Med Hyg, 2021. **106**(2): p. 729-731.
34. Dacombe, R.J., et al., *Time delays between patient and laboratory selectively affect accuracy of helminth diagnosis.* Trans R Soc Trop Med Hyg, 2007. **101**(2): p. 140-5.
35. Sartorius, B., et al., *Prevalence and intensity of soil-transmitted helminth infections of children in sub-Saharan Africa, 2000-18: a geospatial analysis.* Lancet Glob Health, 2021. **9**(1): p. e52-e60.
36. Campbell, S.J., et al., *A Critical Appraisal of Control Strategies for Soil-Transmitted Helminths.* Trends in Parasitology, 2016. **32**(2): p. 97-107.

37. Katz, N., A. Chaves, and J. Pellegrino, *A simple device for quantitative stool thick-smear technique in Schistosomiasis mansoni.* Rev Inst Med Trop Sao Paulo, 1972. **14**(6): p. 397-400.
38. Nikolay, B., S.J. Brooker, and R.L. Pullan, *Sensitivity of diagnostic tests for human soil-transmitted helminth infections: a meta-analysis in the absence of a true gold standard.* International Journal for Parasitology, 2014. **44**(11): p. 765-774.
39. Montresor, A., *Arithmetic or geometric means of eggs per gram are not appropriate indicators to estimate the impact of control measures in helminth infections.* Trans R Soc Trop Med Hyg, 2007. **101**(8): p. 773-6.

Click here to access/download
Supporting Information
PRISMA Checklist STH.docx

Table of included and excluded studies

Click here to access/download
Supporting Information
STH - Included excluded table.xlsx
