# Supplementary material for: The impact of community based interventions for the prevention and control of soil-transmitted helminths: A systematic review and meta-analysis
Source: PLOS Glob Public Health. 2024 Oct 10;4(10):e0003717. doi: 10.1371/journal.pgph.0003717 (PMC11466416; doi:10.1371/journal.pgph.0003717)
Supplement: Attachment — Submitted filename: Response to reviewers table_LB.docx [file pgph.0003717.s006.docx]

We are deeply grateful for your time and thoughtful feedback. Your comments and suggestions have been informative and have helped us improve the quality of our work. Our responses to your comments are outlined below. Page and line numbers refer to the MS Word numbering on the document with tracked changes.

# Reviewer 1 comments

The manuscript has undergone comprehensive revisions, with significant enhancements made by the authors to improve its overall quality. The comments have been effectively addressed, resulting in a more comprehensive search strategy, and improved results.

|  | **Reviewer 1** | **Response** | **Action taken** |
| --- | --- | --- | --- |
|  | Reviewer’s comment |  |  |
| 1 | It would be standard practice to exclude non randomized or pseudorandomized trials from such a review. The inclusion of other study designs introduces potential bias and is difficult to interpret. Would suggest the primary analysis should only include RCTs. | Revised accordingly. | We have opted to exclude non-randomised studies from the meta-analysis. Other study designs are included in the narrative synthesis to ensure comprehensive contextual understanding and insight into the range of available data. |
| 2 | It is not at all clear why CBIs would be lumped together for such a review. There is no real justification for why one would choose to group such disparate interventions together. Given the huge heterogeneity – which makes interpretation of the meta-analysis pretty much impossible, this is a huge issue. Without adequate justification, there is not a compelling sense that these should be combined. | Revised accordingly. | Page 10 -15  We agree that pooling different intervention types together does make it difficult to interpret the meta-analysis. As a correction, we have separated intervention types within the meta-analysis. Intervention types have been separated into preventive chemotherapy, and Water Sanitation and Hygiene and Health Education. |
| 3 | The outcome (prevalence and intensity of STH) is highly dependent on the sampled population. It is critical to separate studies where the population sampled is among individuals treated vs. the whole population. For example, school based delivery that only samples school going children should not be included together with school based delivery in which the whole population (including adults is sampled). Likewise, community based delivery that measures prevalence in the entire population should not be included with prevalence estimated in children. | Revised accordingly. | Thank you for your insightful comment. We agree that prevalence is highly dependent on the sampled population. Studies that report on community wide treatment either sample all available population in the community or randomly select members of a household within the community with no age restriction.  Studies that report school-based delivery either sample all eligible students in a school or a random sample from the school.  There were no cases where school-based delivery reported results from the whole population (including adults). |
| 4 | Note that the abstract needs updating – some STH are acquired from larvae in soil. In addition to MDA, hygiene and sanitation (WASH) programs are also implemented. | Revised accordingly. | Revised as follows  Page 1, Line 15-16  “…transmitted by eggs released in faeces or when hookworm larvae penetrate the skin.”  Page 1, Line 38-40  We also mention studies reporting the impact of WASH programs in the control of STH in the conclusion section of the abstract. |
| 5 | Please clarify whether LF program data were included – and if so, note those studies separately. | Thank you for this comment. Although LF and STH are commonly investigated together in some studies, LF is not considered a STH. Therefore LF program data was not included in this review. | - |
| 6 | Please note that WHO guidelines encourage STARTING MDA if prevalence exceeds 20% but once started it should be continued even when prevalence is lower. | Thank you for this comment. We made reference to this point in the third paragraph of our introduction section. | Page 2, Line 62-64  The sentence reads: “Regular preventive chemotherapy (PC) with the anthelminthic drugs albendazole or mebendazole is recommended by the WHO in areas where prevalence exceeds 20%” |
| 7 | Why did the authors limit to SSA? There are many Asian studies that could have been included. There is no solid rationale provided. | Thank you for your comment. We have revised accordingly. | We had limited to SSA to focus the study and because SSA is disproportionately affected compared to other regions. However, we have now revised the search strategy and removed the SSA search terms. The new studies are now included in our review. |
| 8 | Please describe the differences in Kato-Katz methods between studies – perhaps include in table. Time to read, QA/QC, etc. This has an enormous impact on hookworm detection in particular. | Revised accordingly | Page 8-9  We have included another table (Table 2) in the section describing the characteristics of included studies. This table describes the testing methods used by the included studies. |
| 9 | Figure 1 (flowchart) should be updated with more information – for example, why were 8 not retrieved? What is RoB? | Revised accordingly | Page 4-5  We have reviewed that flowchart and added more information as suggested. RoB- risk of bias, this has been written out in full. |
| 10 | Table 1 Njenga et al 2014 – why is Quality listed as PC? | Revised accordingly | Page 5  Thank you for spotting this error in the manuscript, which should read ‘Poor’ not ‘PC’. We have corrected this in our revision. |
| 11 | Suggest age stratified prevalence and intensity be reported. | Thank you for your suggestion regarding age stratification in reporting prevalence and intensity. Regrettably, the studies included in our systematic review did not uniformly provide age-stratified data for prevalence and intensity measures. | Page 18, Line 457-460  While we aimed to extract as much detailed data as possible from the included studies, the absence of consistent age-specific reporting in the primary literature limited our ability to provide a comprehensive age-stratified analysis in our review. However, we did note this limitation in our manuscript to ensure transparency regarding the available data's scope and potential gaps. |
| 12 | The comparison of six monthly versus annual tx must be adjusted for baseline prevalence (or stratified by prevalence) as guidelines recommend frequency dependent on baseline prevalence. | We agree that comparison of six monthly versus annual treatment should be adjusted for baseline prevalence. We would like to point out that included studies reporting on semi-annual vs. annual had comparable baseline prevalence which allows us to compare both treatment frequencies. | - |
| 13 | The comparisons of community to school based delivery are limited by wide confidence intervals – this should be discussed. | Revised accordingly | Page 18, Line 449-456  We agree with you and we have added a paragraph to the discussion highlighting this point. |
| 14 | More discussion regarding the available lack of evidence for the benefit of WASH interventions for STH is needed. | Revised accordingly | Page 17, Line 392-397  We have included a paragraph specifically highlighting the available evidence gaps concerning the efficacy/ effectiveness of WASH interventions for STH. We have underscored the limitations in the existing literature, highlighting the scarcity of robust data directly demonstrating the impact of WASH interventions on STH. |

# Reviewer 2 comments

|  | **Reviewer 2** | **Response** | **Action taken** |
| --- | --- | --- | --- |
|  | Reviewer’s comment |  |  |
| 1 | Regarding data availability, it seems the search terms that the authors indicated having been uploaded cannot be found in the submission package. Can the authors check if it is uploaded successfully at the revision phase? | We will ensure complete submission on revision. | - |
| 2 | It is a little challenging to understand what this pronoun refers to at first read. So, replacing it with “prevalence” may be better. | Revised accordingly. | Revised as follows  Page 2, Line 82  “... semi-annual PC in communities where prevalence exceeds 50% [9].” |
| 3 | “NTDs”? | Corrected the typo. | Page 2, Line 87  It has been corrected to NTDs |
| 4 | Since the reduction was only observed in one area (among the three areas studied) in the cited article, it seems using a singular form here would be more appropriate. | Revised the sentence. | Revised as follows  Page 2, Line 96  “... efficacy in an area with high drug pressure [11].” |
| 5 | Is it possible to specify here or in the last paragraph of Introduction for innovations/significances of this work by delineating the difference between this and the previous systenatic reivew? The current framing of the specific aim may make some readers think this is just an replication of the previous work, which seems to be a little lackluster. | This is an update on a previous review and aims to strengthen the findings by incorporating the latest evidence, shedding light on the evolving landscape of community-based interventions. | Added the following sentences.  Page 2, Line 101-103  “This review aims to synthesise the latest evidence, shedding light on the evolving landscape of community-based interventions.” |
| 6 | Would “efficacy/effectiveness” be a more appropriate descriptor here if the eligible interventions included both controlled and uncontrolled studies? | Page 3, line 120  We use effectiveness in the manuscript. | - |
| 7 | Comment attached to manuscript: Can the authors justify why other at-risk populations in the Americas and East Asia, mentioned in Introduction, were not included in this reivew? | Revised accordingly | We had limited to SSA to focus the study and because SSA is disproportionately affected compared to other regions. However, following your comment and those of other reviewers, we agree that this rationale is not solid enough. Therefore, we have revised the search strategy and removed the terms limiting the search to SSA. The new studies are now included in our review. |
| 8 | Since the 3rd author has been mentioned previosuly, “the” would be more appropriate here. | Revised the sentence | Revised as follows  Page 3, Line 143  “... discrepancies were resolved with the third author (LB).” |
| 9 | It seem growth indices are part of nutritional outcomes, so “such as” may be better here. Since only data for anemia were available, the authors may consider adjusting the writing on secondary outcome here to focus on anemia. When growth outcomes like stunting are mentioned in Method, people ususally expect seeing related data in Result. | Revised accordingly. | We agree with your comment. We have added more studies with nutritional indices and the sentence now reads correctly. |
| 10 | The authors need to reference Figure 1 here. | Revised accordingly | Revised as follows  Page 4, Line 184  “... presents further details (Figure 1).” |
| 11 | Can the authors present these abbreviations in their full forms in the Figure? | Revised accordingly | We have written the abbreviations in full. |
| 12 | Is “on” missed here? | Correction made | Page 5, line 220  “...focussed on community-based drug treatments.” |
| 13 | Since Tables may be read independently from a manuscript, can the authors provide a foot note to delineate the full forms of the abbreviations used in this table? | Revised accordingly | We opted to write out abbreviations in full. |
| 14 | The use of “N” seems ubiquitious in the below column, which may be simplified by removing them below and change the column name to something like “Sample size, age range”. Please do use comma to separate the two components if proceed with this change. | Revised accordingly | Page 5-8  We agree with you and we have revised the column accordingly. |
| 15 | What is this category? Is this a typo? | Correction made | Page 5  PC corrected to “poor” |
| 16 | Can these regions be specified? | Revised accordingly | All regions in Table 1 have been listed in full |
| 17 | Can the authors strengthen the footnotes here? Seemingly, they are in different categories and not self-explanatory. Also, can the authors adjust the footnotes to show the text above that are blocked? | Revised accordingly | Figure 2, page 11  We have revised the footnotes to be more explanatory. We have also added a new image with all sections appearing fully |
| 18 | Can the authors replace the abbreviations in Figures 2 and 3 and their captions with their full forms, as figures may be accessed by readers independently from the manuscript? | Revised accordingly | We appreciate that figures may be accessed independently. We have written out all abbreviations in full. |
| 19 | Please consider adding “et al.” here as this manuscript has multiple authors. | The section of the work has been revised | Page 14, line 299  Paragraph revised. |
| 20 | Can the authors add “statistically significant” here? Seemingly, the point estimates between the two still differ despite being not significant. | Revised accordingly | Page 15, line 324  “...shows no statistically significant difference in prevalence reduction...” |
| 21 | Similar to my earlier comments, the authors may consider providing full forms of the abbreviations mentioned in Tables 2 and 3 using a footnote for each table. | Revised accordingly | We have written out all abbreviations in full. |
| 22 | The I^2 in Tables 2 and 3 seems ubiquitious and can be simplified by moving it to the heading of each table. | Revised accordingly | Page 15  The I^2 in Tables 2 and 3 have been moved to the heading. |
| 23 | Can the authors articulate this sentence by indicating the outcome(s) targeted by the treatment delivery? | We have rephrased the sentence to ensure clarity. | Page 15, line 335-337  “However, there is no statistically significant difference in outcomes between both treatment delivery methods.” |
| 24 | The authors may add a comma prior to this to make the sentence more easily followed. | Revised accordingly | Correction in Page 16, line 372 |
| 25 | The authors may consider adding “relative” here, as seemingly by absolute percentage difference hookworm fell in a greater magnitude. | Revised accordingly | Page 16, line 383  “...the relative prevalence of hookworm fell...” |
| 26 | It seems “could” would be better here as things were discussed retrospectively here. | The sentence was no longer relevant given the revisions to the meta-analysis | Page 18, line 464 - 466 |
| 27 | Is this a future direction or something that could have been conducted in this study? Please clarify.  Can the authors explain why the analyses mentioned in liminitation that could have been done ware not conducted?  Can the authors explain why this was not being undertaken? | While our review focused specifically on pooling prevalence and nutritional data, the idea of a meta-regression presents an intriguing pathway for future investigations. We intend to explore meta-regression as well as outcomes such as re-infection intensity, changes in infection intensity categories (e.g., very high to high), cognitive development, and behavioural outcomes in a future project. | - |

# Reviewer 3 comments

|  | **Reviewer 3** | **Response** | **Action taken** |
| --- | --- | --- | --- |
|  | Reviewer’s comment |  |  |
| 1 | I don't think you can pool prevalence from different kinds of interventions (unless you did some modeling and accounted for heterogeneity). What's only common among them is that they're "community-based" and these interventions would be very different from each other. Perhaps you can pool “endline” prevalence from the similar interventions. | We agree that the high level of heterogeneity in the studies does not allow pooling of different interventions. We have adopted your suggestion and pooled only “endline” prevalence from the similar interventions. | - |
| 2 | CBI definition is strange – why combine school-based interventions with community-wide treatment? | Although not consistently defined in literature, CBIs include non-clinical interventions targeted at individuals within the communities where they live. This can include School-based and Community-wide treatment. This also follows the definition of the previous review on this topic. However, we agree that they should be analysed seperately. We have revised our analysis accordingly. | - |
| 3 | Underlying data that allowed the calculation should be included as supplement for transparency. The data should be detailed enough to allow reproducibility | We have included the primary outcome data extracted as supplement. | *-* |
| 4 | The search was made more than a year ago – please update | We have updated the search to Nov 2023. |  |
| 5 | Too many abbreviations – limit it to minimum, perhaps no more than 5 abbreviations. | We have tried to limit the use of abbreviations as much as possible. We have limited their use to only well known abbreviations which are commonly used in STH literature. | - |
| 6 | Include search strings either as a table or a supplement | We have included the search string as supplement. | - |
| 7 | Reasons for exclusion of records (n = 2,269) should be included. This 2269 should be broken down. Reasons for records not retrieved should be indicated. Actually, for each step in the PRISMA flow diagram, the reasons should be broken down. | Thank you for this suggestion. We have revised the PRISMA flowchart to properly indicate the reasons for exclusion. | Page 4- 5  PRISMA flowchart revised |
| 8 | Table 1 – why is species just STH, I thought we’re not combining? Include number of clusters | Thank you for pointing out this error. We have made the correction and listed the species in the revision. Please note that hookworm species (*Ancylostoma duodenale* and *Necator americanus*) are reported as ‘hookworm’ in most studies. We refer to both species as ‘hookworm’ in our review and do not exclude studies that do the same. | - |
| 9 | For all tables - All abbreviations in the table should be spelled out below the table. | We have spelled out all abbreviations below the table. | - |
| 10 | Please proof read – why is there an intervention “school-based” and quality as “PC” Should be explained below the table reason for quality | This is a typo, it should read ‘Poor’ not ‘PC’. We have corrected the typo in our revision. | Page 5  PC corrected to “poor” |
| 11 | This is really not just an update from Salam since yours only covered sub-Saharan Africa | We have revised the search strategy to remove the limitation to sub-Saharan Africa. | - |
