# Supplementary material for: The impact of community based interventions for the prevention and control of soil-transmitted helminths: A systematic review and meta-analysis
Source: PLOS Glob Public Health. 2024 Oct 10;4(10):e0003717. doi: 10.1371/journal.pgph.0003717 (PMC11466416; doi:10.1371/journal.pgph.0003717)
Supplement: Attachment — Submitted filename: Response to reviewers table_24_07_2024.doc [file pgph.0003717.s007.doc]

We are deeply grateful for your time and thoughtful feedback. Your comments and suggestions have been informative and have helped us improve the quality of our work. Our responses to your comments are outlined below. Page and line numbers refer to the MS Word numbering on the document with tracked changes.

# Reviewer 1 comments

The authors have done a very commendable job in responding to the reviewer comments and the paper is much improved. I believe that the current manuscript will be a valuable addition to the existing literature.A few minor concerns that remain;

|  | **Reviewer 1** | **Response** | **Action taken** |
| --- | --- | --- | --- |
|  | Reviewer’s comment |  |  |
| 1 | The forest plots suggest that with the exception of trichuris, the CI of the pooled estimate of effect crosses zero, suggesting no significant impact on either prevalence or intensity. The narrative of the results and discussion should be updated to reflect this. | Revised accordingly. | Thank you for your insightful comment. We have updated the narrative of the result and discussion sections to address your comment. The revisions can be seen on Page 8 and Page 11. |
| 2 | Given the above lack of significant effect, I am not clear that a subgroup analysis is warranted? | Thank you for your insightful comment regarding the subgroup analysis. We appreciate your concerns about the significance of the effects observed. However, we believe that subgroup analysis remains crucial for several reasons:   1. Community-based interventions are often planned and implemented in diverse ways depending on local context, resources, and target populations. Subgroup analysis allows us to explore how these variations impact the effectiveness of interventions.Even if the overall effect appears non-significant, there may be subgroups where the intervention is particularly effective or ineffective. Identifying these differential effects can help refine and improve intervention strategies. 2. The value of subgroup analysis also lies in documenting and evaluating the impact of standard ways that interventions have been planned and implemented. This may be relevant to policymakers and program developers. | - |
| 3 | Available data suggest that the efficacy for existing PC drugs (albendazole and mebendazole) are LEAST effective for Trichuris - yet this is where the greatest impact appears to be seen. This is counterintuitive and deserves discussion | Revised accordingly. | Thank you for this insightful comment. We agree with your observation. We have included a discussion of this point in the manuscrip. The revision can be seen on Page 11, lines 319 -333 |

# Reviewer 2 comments

I appreciate the authors' thorough revision based on the reviewers' comments.

|  | **Reviewer 2** | **Response** | **Action taken** |
| --- | --- | --- | --- |
|  | Reviewer’s comment |  |  |
| 4 | Under "Declarations", it seems the quotation mark in this sentence should be removed - The authors declare that they have no competing interests" in this section. | The section of the work has been revised | Quotation mark removed.  Correction on Page 13, line 450 |
